# The effect of emotional security on depressive tendencies in junior high school students: mediation and intervention of interpersonal trust

**DOI:** 10.3389/fpsyg.2025.1736608

**Published:** 2026-01-28

**Authors:** Xinyue Li, Xueqing Chen, Mengmeng Xie, Minghao Zhang

**Affiliations:** 1 College of Education, Institute for Education and Treatment of Problematic Youth, Ludong University, Yantai, China; 2 Hangzhou Dianzi University Information Engineering University, Hangzhou, China; 3 Shandong Provincial Institute of Educational Sciences, Jinan, China

**Keywords:** adolescent, depression tendency, emotional security, family sandplay, interpersonal trust

## Abstract

**Introduction:**

Emotional security is closely linked to adolescents’ mental health, yet the mechanisms through which it relates to depressive tendencies remain unclear. This study examined whether interpersonal trust mediates the association between junior high school students’ emotional security and depressive tendencies, and whether box therapy can enhance interpersonal trust and reduce depression-related symptoms.

**Methods:**

Study 1 administered the Emotional Security Scale, the Parental Peer Attachment Scale, and the Depression Scale to 436 students from a junior high school. Study 2 employed a single-subject experimental design, delivering 16 individual box therapy sessions and 8 family box therapy sessions.

**Results:**

Emotional security, interpersonal trust, and depressive tendencies were significantly correlated. Emotional security was negatively associated with depressive tendencies, and interpersonal trust partially mediated the relationship between emotional security and depressive tendencies. In Study 2, the combined individual and family box therapy intervention improved adolescents’ interpersonal trust and showed a beneficial effect in reducing depressive tendency levels.

**Discussion:**

Interpersonal trust appears to be a key psychological pathway linking emotional security to depressive tendencies among junior high school students. Box therapy, particularly when integrating individual and family sessions, may be a feasible and practical approach to strengthening interpersonal trust and supporting depression-related symptom reduction in this population.

## 1 Introduction

Subthreshold depression, also known as depressive tendency, refers to a depressive state between normal depressive emotions and clinical depression. Individuals with depressive tendencies often experience persistent negative emotions that affect their physical and mental health as well as daily functioning. Without timely identification and intervention, such tendencies may eventually develop into clinical depression (Kou et al., 2022). Early adolescents in junior high school experience dramatic physiological and psychological changes accompanied by strong emotional fluctuations. Depressive tendencies during this period not only impair their normal interpersonal functioning but also hinder their academic and psychological development (Jiang et al., 2021). According to *The Chinese Blue Book of National Depression (2022)*, the onset of depression has shown a trend toward younger ages, with individuals under 18 accounting for 30% of all cases and the prevalence of depression among adolescents reaching 15–20%. Therefore, early screening and intervention for depressive tendencies in junior high school students are essential tasks for school- and community-based mental health services.

Emotional security refers to the emotional experiences individuals have when perceiving interparental conflict (either through direct observation or recollection) and is considered a key psychological outcome of family relationships. According to ecological systems theory, family relationships serve as the core of the microsystem and exert a profound influence on adolescents’ psychological development (Bronfenbrenner, 1979). Emotional security is an important dimension for measuring family relationships (Hoegler et al., 2023). Previous studies have shown that adolescents who lack emotional security regarding their parents’ relationship are more likely to develop psychological problems such as social anxiety and depression (Davies et al., 2006). Davies and Martin (2013) further demonstrated that lower levels of emotional security may lead to hostility and suspicion toward others, increasing the risk of depressive disorders and hindering social adaptation in adolescence (Davies and Martin, 2013). From an attachment perspective, repeated exposure to insecurity in the family context may shape adolescents’ internal working models of the self and others, which can in turn influence expectations about others’ availability and reliability in later relationships (Bowlby, 1999).

At the same time, during the junior high school stage, adolescents’ intimate relationships shift from parents to peers. This developmental period is critical for forming self-identity, pursuing autonomy, and developing an independent sense of self. Difficulties in managing relationships with parents or peers during this stage can weaken social support systems, hinder the establishment of positive interpersonal relationships, and consequently increase depressive symptoms. Chinese studies have found that crises of interpersonal trust can lead to lower emotional security, reduced subjective wellbeing, and even depression (Li et al., 2025). Moreover, greater interpersonal stress is associated with higher and more persistent levels of depression (Zhao et al., 2025). Thus, depression caused by interpersonal difficulties or lack of interpersonal trust constitutes a major proportion of adolescent depression cases (Nakano et al., 2022).

Interpersonal trust refers to a generalized expectancy that others, words, promises, and statements can be relied upon (Rotter, 1967). In addition, trust has been conceptualized as a willingness to be vulnerable based on positive expectations of another’s trustworthiness, which is often discussed in terms of perceived ability, benevolence, and integrity (Mayer et al., 1995). Prior research has identified several factors influencing adolescents’ interpersonal trust, including attachment relationships (Bao et al., 2022; Armsden and Greenberg, 1987) and sociocultural contexts (Pellegrini et al., 2021). Bowlby et al. (1992) conceptualized emotional security in parent–child relationships as the perceived availability of attachment figures and susceptibility to fear. Building on this perspective, many studies have examined the predictive effect of emotional security on interpersonal trust (Manqing and Zhe, 2024). Empirical evidence suggests that individuals with higher levels of psychological security tend to cope with interpersonal problems more positively and exhibit stronger interpersonal trust (Mitterer and Mitterer, 2023). Therefore, emotional security may influence adolescents’ depressive tendencies indirectly by shaping their levels of interpersonal trust.

Sandplay therapy is a nonverbal psychotherapeutic approach in which clients freely select miniature figures and create scenes in a sand tray under the therapist’s guidance, thereby expressing their inner world (Zhang, 2012). Previous studies have shown that sandplay therapy can enhance clients’ interpersonal trust by improving communication and stress-coping styles (Yu and Zhan, 2021). However, the exploration of its specific intervention mechanisms remains limited, particularly regarding its underlying processes, population applicability, and long-term effects. Existing evidence indicates that group sandplay therapy produces greater improvements in multiple dimensions of interpersonal trust among college students compared to individual sandplay (Yu and Zhan, 2021). Moreover, dynamic changes in clients’ interpersonal trust and depressive tendencies during sandplay interventions can be observed and evaluated through the frequency of symbolically meaningful toys and the occurrence of positive linguistic expressions (Zhang, 2012). Given the effectiveness of sandplay therapy in enhancing interpersonal trust, the present study integrates both family and individual sandplay approaches to reduce depressive symptoms among junior high school students by improving their levels of interpersonal trust.

Although prior studies have linked family-related security (or attachment-related constructs) to adolescent depressive symptoms and have separately documented associations between interpersonal trust and depression, three gaps remain. First, the mechanistic pathway through which family-based emotional security is translated into depressive tendencies via adolescents’ relational expectations (i.e., interpersonal trust) has been less explicitly tested in junior high school populations. Second, existing work is often limited to correlational demonstrations without integrating process-oriented evidence that can illuminate how trust-related meanings and interaction patterns change during intervention. Third, school-based research rarely examines a combined individual–family approach that targets both intrapersonal emotional processing and family interaction patterns. The present two-study research addresses these gaps by (a) testing interpersonal trust as a partial mediator between emotional security and depressive tendencies in a large school sample (Study 1) and (b) using a single-subject ABA design to provide preliminary, phase-based process evidence from a combined individual and family sandplay protocol implemented in a school counseling context (Study 2).

### 1.1 Research questions and hypotheses

Based on the above rationale and identified gaps, the present study addressed the following research questions and hypotheses.

#### 1.1.1 Study 1 (survey study): hypotheses

*H1*:    Emotional security will be negatively associated with depressive tendencies among junior high school students.
*H2*:    Emotional security will be positively associated with interpersonal trust, and interpersonal trust will be negatively associated with depressive tendencies.
*H3*:    Interpersonal trust will partially mediate the association between emotional security and depressive tendencies.

#### 1.1.2 Study 2 (single-case ABA intervention): hypotheses

*H4*:    Across the ABA phases, case-level improvements in interpersonal trust and reductions in depressive tendencies will be observed following the combined individual and family sandplay protocol.
*H5*:    Process indicators in sandtray productions and family interaction patterns (e.g., trust/communication-related themes, increased integration, reduced fragmentation) will show phase-related changes consistent with improvements in interpersonal trust and reductions in depressive tendencies.

In sum, the present work advances the literature by (1) testing a theoretically motivated mediation pathway in a large school sample, (2) complementing correlational evidence with phase-based case evidence, and (3) evaluating a combined individual–family sandplay protocol in a school-based context.

## 2 Study 1: the relationship among emotional security, interpersonal trust, and depressive tendencies in junior high school students

### 2.1 Participants

Using a cluster sampling method, questionnaires were administered to students from Grades 6 to 8 at a junior high school. The survey included the Emotional Security Scale, the revised Inventory of Parent and Peer Attachment (IPPA-R), and the Center for Epidemiologic Studies Depression Scale (CES-D). A total of 450 questionnaires were distributed, and 446 were returned. After removing 10 incomplete responses, 436 valid questionnaires were retained, yielding an effective response rate of 96.89%. Among the participants, 219 were boys and 217 were girls; 148 were in Grade 6, 142 in Grade 7, and 146 in Grade 8.

The study protocol was approved by the Institutional Review Board of Ludong University (Approval No. LDU-IRB2025140007) and complied with the Declaration of Helsinki. Written informed consent was obtained from all participants and their custodial authorities prior to data collection. Participants were informed that participation was voluntary and that they could withdraw at any time without penalty. To protect confidentiality, all data were de-identified using unique codes, stored on password-protected devices, and accessed only by the research team; any potentially identifying information was removed from reports. Given the assessment of depressive symptoms, a risk-management procedure was implemented: students indicating elevated depressive severity or potential self-harm risk were promptly referred to the school mental health professional and, when necessary, their guardians were notified in accordance with school policy and safeguarding requirements.

### 2.2 Measures

#### 2.2.1 Emotional security scale (SIS)

Emotional security was assessed using the Security in the Interparental Subsystem (SIS) developed by Davies and Martin (2013). The scale consists of two factors—Emotional Reactivity (9 items, e.g., “When my parents argue, I feel sad, scared, or angry”) and Negative Representation (8 items). All items were rated on a 4-point scale, with higher scores indicating poorer perceived emotional security. In this study, the Cronbach’s α coefficient for the total scale was 0.92, and for the subscales: Emotional Reactivity α = 0.89, Negative Representation α = 0.86.

#### 2.2.2 Revised inventory of parent and peer attachment (IPPA-R)

The IPPA-R contains three subscales—Father Attachment, Mother Attachment, and Peer Attachment—each comprising three dimensions: Trust, Communication, and Alienation (Armsden and Greenberg, 1987). This study focused on the Trust dimension, which includes trust in father, mother, and peers (30 items in total). Responses were rated on a 5-point Likert scale. Scores between 1 and 17 indicate low trust, 18–34 represent moderate trust, and scores above 35 reflect high trust. Higher scores indicate stronger interpersonal trust. The overall Cronbach’s α of the scale was 0.93, and for each dimension: Mother Trust α = 0.89, Father Trust α = 0.90, and Peer Trust α = 0.83.

#### 2.2.3 Center for epidemiologic studies depression scale

Depressive tendencies were measured using the Center for Epidemiologic Studies Depression Scale (CES-D) developed by Radloff (1977). The scale consists of 20 items rated on a 4-point Likert scale, with higher scores indicating greater frequency of depressive symptoms. In the present study, the Cronbach’s α coefficient of the scale was 0.89.

### 2.3 Data analysis

All data were coded and analyzed using SPSS 22.0. Statistical analyses included reliability testing, common method bias assessment, and correlation analyses among emotional security, interpersonal trust, and depressive tendencies.

### 2.4 Results

#### 2.4.1 Common method bias test

To control for potential common method bias, Harman’s single-factor test was conducted (Zhou and Long, 2004). The results showed that the first unrotated factor accounted for 24.56% of the total variance, which is below the critical threshold of 40%, indicating that no significant common method bias existed in this study.

#### 2.4.2 Correlations among emotional security, interpersonal trust, and depressive tendencies

Pearson’s correlation analyses were conducted to examine the relationships among emotional security, interpersonal trust, and depressive tendencies (see Table 1). The results indicated that all variables and their dimensions were significantly correlated with each other.

**TABLE 1:** Correlation matrix among depressive tendencies, emotional security, and interpersonal trust (*N* = 436).

Variables	1 Emotional security	2 Emotional reactivity	3 Negative representation	4 Maternal trust	5 Paternal trust	6 Paternal trust	7 Interpersonal trust	8 Depressive tendency
1	1							
2	0.94^**^	1						
3	0.92^**^	0.73^**^	1					
4	−0.27^**^	−0.22^**^	−0.28^**^	1				
5	−0.28^**^	−0.23^**^	−0.30^**^	0.66^**^	1			
6	−0.14^**^	−0.08	−0.18^**^	0.46^**^	0.44^**^	1		
7	−0.28^**^	−0.22^**^	−0.31^**^	0.86^**^	0.87^**^	0.74^**^	1	
8	0.48^**^	0.43^**^	0.46^**^	−0.48^**^	−0.42^**^	−0.33^**^	−0.50^**^	1
*M*	35.53	19.39	16.14	39.12	38.72	37.62	115.45	34.26
*SD*	11.65	6.57	5.93	8.52	9.19	7.48	20.86	10.54

Emotional Security includes two subdimensions: Emotional Reactivity and Negative Representation. Interpersonal Trust is composed of Maternal Trust, Paternal Trust, and Peer Trust.

**p < 0.01, the same applies to all subsequent tables.

#### 2.4.3 Test of the mediating effect of interpersonal trust

First, Model 4 of Hayes’s PROCESS macro for SPSS (Hayes, 2013) was used to test the mediating role of interpersonal trust in the relationship between emotional security and depressive tendency. As shown in Table 2, emotional security significantly and positively predicted depressive tendency, while it significantly and negatively predicted interpersonal trust. When interpersonal trust and emotional security were entered simultaneously into the regression model, interpersonal trust was significantly and negatively associated with depressive tendency, and emotional security remained a significant negative correlate of depressive tendency.

**TABLE 2:** Test of the mediating effect of interpersonal trust.

Regression model		Model fit			Significance	
Outcome variable	Predictor variable	*R*	*R* ^2^	*F*	β	*t*
Depressive tendency		0.49	0.24	44.38		
	Emotional security				0.48	11.17^***^
Interpersonal trust		0.30	0.09	14.42		
	Emotional security				−0.29	−6.25^***^
Depressive tendency		0.61	0.38	64.99		
	Emotional security				0.36	8.99^***^
	Interpersonal trust				−0.39	−9.86^***^

***p < 0.001.

The mediation analysis further revealed that the 95% bootstrap confidence interval for the direct effect of emotional security on depressive tendency did not include zero, and the 95% bootstrap confidence interval for the indirect effect via interpersonal trust also excluded zero. These results indicate that interpersonal trust partially mediates the relationship between emotional security and depressive tendency (see Table 3). The indirect effect accounted for 22.92% of the total effect.

**TABLE 3:** Decomposition of total, direct, and indirect effects.

Effect type	Effect	Boot SE	Boot LLCI	Boot ULCI
Indirect effects	0.11	0. 03	0.06	0.18
Direct effects	0.36	0.04	0.28	0.44
Total effects	0.48	0.04	0.39	0.56

Boot SE, bootstrap standard error; Boot LLCI, lower limit of the 95% bootstrap confidence interval; Boot ULCI, upper limit of the 95% bootstrap confidence interval.

## 3 Study 2: sandplay intervention on interpersonal trust among junior high school students

### 3.1 Participants

Based on the questionnaire results from Study 1, six participants with interpersonal trust scores below 35 and depressive tendency scores above 20 were screened. With the consent of the participants and their families, one 14-year-old female student (referred to as Participant A) and her parents were selected for the intervention study. Study 2 was conducted under IRB approval from Ludong University (Approval No. LDU-IRB2025140007). One student and the student’s parents (family sessions) provided written parental consent and student assent; participation was voluntary and families could withdraw at any time. Data and sandtray records were de-identified and securely stored with restricted access. Depressive-symptom risk was monitored, and any safety concerns prompted referral to school mental health services and supervisor consultation, with guardian notification per school policy.

Participant A was an only child in the third year of junior high school. Her parents frequently argued, and these conflicts often involved her. She exhibited strong feelings of self-loathing and guilt, believing that her parents’ marital discord was her fault and that their lives would improve if she disappeared. During the 2 weeks prior to the intervention, she reported severe insomnia, depressed mood, loss of interest, declining academic performance, and interpersonal difficulties accompanied by school aversion.

### 3.2 Instruments

#### 3.2.1 Revised inventory of parent and peer attachment (IPPA-R)

Same as used in Study 1.

#### 3.2.2 Center for epidemiological studies depression scale (CES-D)

Same as used in Study 1.

#### 3.2.3 Sandplay tools (sand tray, sand, and miniatures)

A standard sandplay therapy set was used, including a tray measuring 57 cm (width) × 72 cm (depth) × 7 cm (height) with blue inner walls, half-filled with clean and soft sand. Approximately 2,000 miniature figures were arranged on two shelves, categorized by type (e.g., people, animals, plants, buildings, vehicles, food, furniture) (Zhang, 2012).

### 3.3 Research design

A single-subject experimental design was adopted (A–B–A format). The first A phase represented the baseline stage (A1), B represented the intervention stage, and the second A represented the follow-up stage (A2).

*Baseline Phase (A1)*: From May 16 to May 23, 2022, the IPPA-R and CES-D were administered twice prior to the intervention to obtain a stable baseline for comparison with post-intervention data.

*Intervention Phase (B)*: From May 28 to July 18, 2022, eight sessions of sandplay therapy were conducted under the guidance of *The Clinical Practice of Sandplay Therapy* (Zhang, 2012). Data were collected after each session. The same measurement tools, time frame, and environment were maintained as in the baseline phase to compare changes in interpersonal trust and depressive tendencies before and after the intervention.

*Follow-up Phase (A2)*: From August 29, 2022, to February 13, 2023, six follow-up assessments were conducted using the same measurement tools. Data collected during this phase were used to examine the sustainability of the intervention effects.

### 3.4 Implementation plan

#### 3.4.1 Therapist qualifications and supervision

The interventionist in this study was a graduate student specializing in counseling and psychotherapy at a university. During the program, the interventionist completed relevant theoretical coursework in psychological counseling, which provided a foundation for the counseling practice implemented in this research. After the research topic was determined, the interventionist conducted systematic self-study of sandplay therapy through professional texts and completed a sandplay training course in July 2021. Since October 2020, the interventionist has served as an assistant counselor at the university’s mental health center. In addition, from March to July 2022, the interventionist completed an internship as a school mental health teacher at the junior high school attended by the participant. During the thesis-writing period, the university mental health center assigned a clinical supervisor who provided guidance and supervision for the interventionist’s counseling process. The interventionist continues to receive ongoing training in sandplay therapy and its theoretical foundations, with the aim of applying this approach in routine mental health classes and school-based counseling services in junior high school settings.

#### 3.4.2 Individual and family sandplay therapy protocol

From May 2022 to February 2023, a total of 16 sessions of individual sandplay therapy and 8 sessions of family sandplay therapy were conducted. The specific procedures, instructions, production rules and precautions during the operation of individual sandplay and family sandplay in this study. The individual box family consultation plan and the family box family implementation plan adopted are respectively shown in Tables 4, 5.

**TABLE 4:** Individual sandplay therapy implementation plan.

Number of sessions	Sandplay theme	Overall sandtray scene and placement of miniatures
Session 1	No specific theme was assigned. As this was the client’s first exposure to the sandtray, they arranged the miniatures freely. A brief preliminary interpretation was conducted to help the client externalize presenting concerns and to establish the therapeutic alliance (baseline phase).	The overall scene was monotonous and bleak, with a weak expression of ego energy. The sense of energy flow was limited, and the composition appeared relatively closed. The selection of miniatures was narrow in variety, and no self-representational figure was present.
Session 2	No specific theme was assigned. The client arranged the miniatures freely. A brief preliminary interpretation was conducted to gather additional information (baseline phase).	The overall scene was relatively monotonous and showed limited vitality, with a weak expression of ego energy. The composition appeared relatively closed, with limited energy flow. Only a small variety of miniatures was used, occupying a relatively small area of the sandtray, and a self-representational figure was present.
Session 3	Theme assigned: recalling an unforgettable past experience. The client was reminded of childhood memories and titled the sandtray “Childlike Fun.”	The scene was monotonous and lacked vitality, with a weak expression of ego energy. The composition appeared relatively closed, with limited energy flow. The variety of miniatures was relatively restricted, occupying a small area of the sandtray, and a self-representational figure was present.
Session 4	No specific theme was assigned. The client arranged the miniatures freely; trauma-related material emerged, and the presenting problems began to surface.	The scene was monotonous, with a relatively closed spatial organization and limited interaction and movement/flow. The composition was fairly concentrated yet occupied only a small proportion of the sandtray, and no self-representational figure was present.
Session 5	Theme assigned: the client’s present self. The client did not give the sandtray work a title.	The scene appeared bleak, with a limited variety of miniatures. Overall, the sense of energy flow was minimal, while the placement area expanded. A self-representational figure was present.
Session 6	No specific theme was assigned. The client arranged the miniatures freely, and changes in the sandtray work were observed.	The scene became richer, with an increased variety of miniatures. Figures and objects representing energy and support emerged, but no self-representational figure was present.
Session 7	No specific theme was assigned. The client arranged the miniatures freely, and the sandtray work began to show signs of counterbalancing forces as well as an expression of ego energy.	The scene was relatively rich, with a wide variety of miniatures, and a self-representational figure was present. During this phase, marked emotional fluctuations were observed.
Session 8	No specific theme was assigned. The client arranged the miniatures freely, and miniatures related to interpersonal trust appeared in the sandtray work.	The scene was relatively monotonous, with a limited variety of miniatures; however, the overall composition appeared harmonious. Positive terms such as “trust” and “communication” emerged, along with corresponding miniature arrangements.
Session 9	No specific theme was assigned, and the client arranged the miniatures freely. The session could be further elaborated by drawing on the client’s interactions with family over the weekend.	The scene was relatively monotonous, with a limited variety of miniatures. However, expressions of ego energy and renewal emerged; the composition began to show vitality and liveliness, and a self-representational figure was present.
Session 10	No specific theme was assigned. The client arranged the miniatures freely, and the session served as an observation of post-intervention effects following the family sandplay therapy.	The scene was relatively rich, with an increased variety of miniatures. Greater movement/flow and vitality were evident in the composition, and a self-representational figure was present.
Session 11	No specific theme was assigned; the client arranged the miniatures freely (follow-up).	The client was able to articulate and design the sandtray work effectively. Although only a small number of miniatures was used, they were arranged in a concentrated manner in the central area. Ego energy appeared to strengthen, and a self-representational figure was present.
Session 12	No specific theme was assigned; the client arranged the miniatures freely (follow-up).	The scene was relatively full. The variety of miniatures increased noticeably, particularly those symbolizing ego energy and positive emotions. A self-representational figure was present.
Session 13	No specific theme was assigned; the client arranged the miniatures freely (follow-up).	Miniatures representing interpersonal communication, interaction, and ego energy increased markedly. The scene became richer overall, and the self-representational figure appeared more substantial, positive, and empowered.
Session 14	No specific theme was assigned; the client arranged the miniatures freely (follow-up).	A relatively large number of miniatures representing ego energy were present, and the scene was richer. The client used more positive words when describing the scene, and the self-representational figure appeared more substantial, positive, and empowered.
Session 15	No specific theme was assigned; the client arranged the miniatures freely (follow-up).	Miniatures representing interpersonal interaction and ego energy increased markedly. The scene was relatively rich, the client used more positive descriptive words, and the self-representational figure appeared more substantial, positive, and empowered.
Session 16	No specific theme was assigned; the client arranged the miniatures freely (follow-up).	Miniatures representing interpersonal communication, interaction, and ego energy were abundant. The scene was full, the client used many positive words when describing it, and the self-representational figure appeared substantial and powerful.

**TABLE 5:** Family sandplay therapy implementation plan.

Number of sessions	Sandplay theme	Overall sandtray scene and placement of miniatures
Session 1	No specific theme was assigned. As this was the family’s first exposure to the sandtray, they arranged the miniatures freely. A brief preliminary interpretation was conducted to help the family externalize presenting concerns and to establish the therapeutic alliance.	Interpersonal interaction was limited. The scene reflected a “working independently” pattern, with placements dispersed across three separate areas and weak connections among them.
Session 2	No specific theme was assigned. The family jointly completed a sandtray creation and, after discussion, titled it “Home.”	Interpersonal interaction remained limited. Interaction between the mother and the child was more frequent, whereas the father continued placing miniatures within his own area. The overall scene was relatively rich.
Session 3	No specific theme was assigned. Problems began to surface and conflicts emerged. After discussion, the theme was identified as “Life,” although the child was not satisfied with this theme.	Interpersonal interaction began to shift, but conflicts emerged. The child started removing the parents’ miniatures, and the overall scene became much fuller and richer than before.
Session 4	No specific theme was assigned. The scene lacked integration, and the child began to show resistance.	Interpersonal interaction increased, accompanied by frequent conflict. There were multiple abrupt and disharmonious elements in the work; however, the overall scene was rich, with a strong sense of movement/flow.
Session 5	No specific theme was assigned. The family jointly completed a sandtray work and titled it “Vacation.” The parents began to listen more attentively to the child’s perspectives and incorporated the child’s ideas.	Interpersonal interaction increased, and conflicts decreased markedly. The father began to adopt a more global perspective, attending to the harmony of the overall scene and providing appropriate adjustments to the child’s placement area. Interaction between the mother and the child remained more frequent than with the father.
Session 6	No specific theme was assigned. The family jointly completed a sandtray work. Family interactions increased, the pace of placement slowed, and members became more focused and engaged. The process entered the healing phase.	Interpersonal interaction increased. Conflicts and the previously fragmented/dispersed pattern were largely absent. The variety of miniatures gradually shifted toward warmer, more energetic elements, and miniatures symbolizing communication increased over time.
Session 7	No specific theme was assigned. The family jointly completed a sandtray work, with increased interaction and gradually improved coordination. Family relationships showed signs of repair.	Interpersonal interaction increased, and the overall composition appeared harmonious. The number and variety of miniatures symbolizing positive meanings increased noticeably, and the scene showed greater integration.
Session 8	Theme assigned: the desired state of the home at present and in the future. Family members’ interactions and relationships were further enhanced.	Interpersonal interaction entered a positive cycle. The overall composition was harmonious; the number and variety of miniatures symbolizing positive meanings increased markedly, and the scene tended toward integration. Family relationships improved substantially.

### 3.5 Results

#### 3.5.1 Effects of sandplay therapy

**FIGURE 1:**
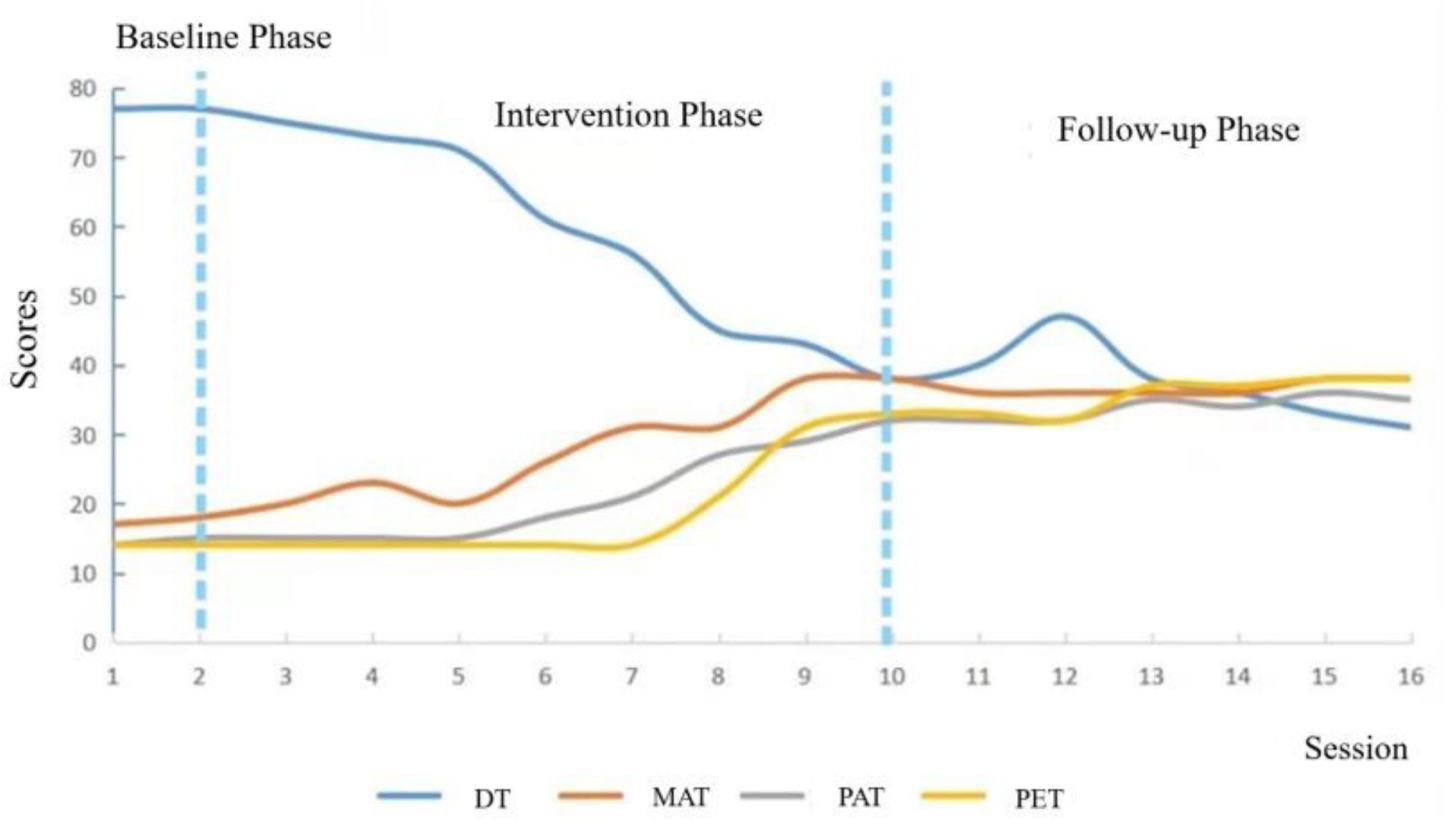
Sandplay intervention process diagram. MAT, Maternal Trust; PAT, Paternal Trust; PET, Peer Trust; DT, Depressive Tendency.

A single-subject experimental design was used for data analysis. The IPPA-R and CES-D were administered in the baseline, intervention, and follow-up phases. Quantitative comparisons of scores before and after intervention are presented in Table 6, and the overall baseline trend is illustrated in Figure 1. The sandbox process is presented in Supplementary Appendix S1.

**TABLE 6:** Scores of each dimension across different assessment phases.

Phase	Session	MAT	PAT	PET	DT
Baseline phase	1	17	14	14	77
Intervention phase	2	18	15	14	77
	3	20	15	14	75
	4	23	15	14	73
	5	20	15	14	71
	6	26	18	14	61
	7	31	21	14	56
	8	31	27	21	45
Follow-up phase	9	38	29	31	43
	10	38	32	33	38
	11	36	32	33	40
	12	36	32	32	47
	13	36	35	37	38
	14	36	34	37	36
	15	38	36	38	33
	16	38	35	38	31

MAT, Maternal Trust; PAT, Paternal Trust; PET, Peer Trust; DT, Depressive Tendency.

#### 3.5.2 Use of toys during the intervention

Toy usage during the intervention sessions was analyzed to identify symbolic representations associated with interpersonal trust and improvement in depressive symptoms. The results indicated that, across both individual and family sandplay sessions, the frequency of toys symbolizing communication, social support, trust, sharing, self-esteem, self-energy, stress coping, renewal, and hope increased as the intervention progressed. This pattern reflected gradual improvements in interpersonal trust and reductions in depressive tendencies.

#### 3.5.3 Frequency of positive lexical expressions

To verify the specificity and mechanism of family sandplay intervention on interpersonal trust, post-session interviews were conducted after each family sandplay activity. The interviews covered self-worth, social support, social skills, and two protective factors against depression—perceived stress and self-efficacy (Isom et al., 2015).

Text coding analysis revealed that the frequency of words related to perceived stress decreased over time, whereas references to self-worth, social support, social skills, and self-efficacy gradually increased, indicating enhanced psychological resilience and interpersonal functioning.

## 4 Discussion

### 4.1 Associations between emotional security, interpersonal trust, and depressive tendencies

This study examined the relationship between emotional security and depressive tendencies among junior high school students and explored the mediating role of interpersonal trust. Results showed that emotional security negatively predicted depressive tendencies, with interpersonal trust serving as a partial mediator.

Consistent with previous findings (Geng et al., 2021), emotional security was a significant negative predictor of depressive tendencies. Emotional security arises from experiences of parental conflict; thus, adolescents exposed to frequent family conflicts are prone to negative emotions and psychological dysregulation (Davies and Cummings, 1998). Persistent exposure to such conflict consumes psychological resources needed for self-regulation, increasing the risk of depression (Jiang et al., 2025). Compared with studies that focus primarily on family conflict frequency or other distal family risk indicators, the current results underscore emotional security as a psychologically proximal factor linked to adolescents’ depressive tendencies, which may help clarify how family climate translates into emotional outcomes.

Interpersonal trust mediated the relationship between emotional security and depressive tendencies. Emotional security positively predicted interpersonal trust, aligning with prior research (Li et al., 2025). According to attachment theory, early emotional connections shape internal working models that influence how individuals perceive self-worth, intimacy, and social interactions (Fearon and Roisman, 2017). Positive family emotional interactions strengthen children’s sense of safety and form the basis of trust toward others (Li et al., 2025). Conversely, repeated family conflicts undermine emotional security, leading to trust crises in social interactions—a pattern confirmed across cultures (Szcześniak et al., 2012). Moreover, interpersonal trust negatively predicted depressive tendencies (Zeng and Xia, 2019). The stress generation model (Hammen, 1991) posits that individuals with depressive vulnerability tend to perceive and respond to stressors passively, reinforcing negative cognitive schemas. Adolescents with low interpersonal trust may avoid social interactions and experience emotional detachment, increasing vulnerability to depression (Jiang et al., 2021). In contrast, those with higher levels of trust can better perceive care and support from others, accumulate psychological resources, and thereby mitigate depressive symptoms (Grace and Schill, 1986). Notably, interpersonal trust functioned as a partial rather than full mediator, suggesting that emotional security may also influence depressive tendencies through additional pathways (e.g., emotion regulation, maladaptive cognitions, or peer-related stress processes). This pattern is consistent with multi-process accounts of adolescent depression and highlights the need to model both relational and intrapersonal mechanisms in future research.

By specifying interpersonal trust as a relational mechanism, the current model moves beyond documenting family risk and identifies a modifiable social–cognitive pathway through which emotional security may shape adolescents’ vulnerability to depressive tendencies. This extends prior research by clarifying not only whether these variables are associated, but also how family emotional climate may be translated into downstream socio-emotional outcomes during early adolescence.

### 4.2 Sandplay intervention on interpersonal trust

Based on toy coding and linguistic analysis (Roesler, 2019; Turner, 2023), changes observed across the ABA phases suggest that the combined individual and family sandplay procedures were associated with improvements in interpersonal trust and reductions in depressive tendencies at the case level. In addition, family sandplay sessions provided observable shifts in interaction patterns that were consistent with improvements in parent–child communication and trust. Given the single-case nature of the design, these findings should be interpreted as preliminary and descriptive rather than conclusive evidence of efficacy. Individual sandplay sessions provided clearer insights into changes in depressive symptoms, whereas family sandplay sessions offered more direct observations of changes in interpersonal trust. This distinction aligns with theoretical perspectives suggesting that individual sandplay better reflects unconscious processes, while group or family sandplay is more effective in observing and improving interpersonal interactions (Turner, 2023). Participant A’s scores for interpersonal trust and depressive tendencies showed a fluctuating but overall positive trend across the baseline, intervention, and follow-up phases—particularly during sessions 3–4 of the intervention and sessions 2–3 of the follow-up. These fluctuations may be associated with stressful life events experienced prior to therapy. From a comparative perspective, this pattern is consistent with clinical process accounts in which emotional activation and symptom fluctuation can occur when distressing material emerges and is processed, before more stable improvements are consolidated.

Therefore, sandplay therapy can serve both as an intervention tool for improving trust and reducing depressive states, and as a preventive, school-based risk assessment tool for relationship and emotional difficulties. In practice, the combined use of individual and family sandplay may be particularly valuable in school settings: individual sessions can facilitate affect expression and self-representation, whereas family sessions can externalize interaction patterns (e.g., fragmentation vs. coordination; conflict vs. cooperation) and provide opportunities to practice communication and trust-building *in vivo*. Nevertheless, further replication with larger samples and controlled designs is required to strengthen generalizability and causal inference.

Relative to prior sandplay studies that focus on either individual or group formats, Study 2 adds value by combining individual and family modalities and documenting phase-based process markers of trust/communication and interactional organization in a school-based setting, thereby providing a more clinically informative picture of how relational change may unfold over time. These case-level process indicators (toy themes and reflection language) provide descriptive evidence that is broadly consistent with the correlational mediation pattern observed in Study 1. However, given the single-case ABA design, this qualitative information should be interpreted as complementary process data rather than confirmatory evidence of efficacy.

### 4.3 Theoretical contributions

The present study contributes to the literature in three ways. First, it integrates family-based emotional security with a relational mechanism—interpersonal trust—to explain depressive tendencies in early adolescence, thereby extending prior work that typically examines these constructs in isolation. Second, the finding of partial mediation suggests a multi-pathway process in which family emotional climate shapes adolescents’ internal sense of safety and simultaneously influences broader social expectations and relational engagement, offering a more process-oriented account of how family experiences translate into emotional outcomes. Third, by combining individual and family sandplay and documenting process markers (e.g., miniature themes related to communication/trust and shifts in spatial organization and interaction), the study provides descriptive evidence on how relational meanings may be externalized and reorganized through symbolic play in a school-based context.

### 4.4 Clinical and practical implications

Practically, the findings suggest several implications for school-based mental health services. First, assessment and intervention efforts may benefit from targeting adolescents’ sense of emotional security and interpersonal trust simultaneously, particularly for students exposed to family conflict. Second, the combined use of individual and family sandplay may be clinically useful: individual sessions can facilitate the expression of internal emotional states, whereas family sessions can make interaction patterns visible and provide opportunities to practice communication and coordination in vivo. Third, the observed process indicators (e.g., emergence of trust/communication-related miniatures, increased integration of the overall scene, and reduced fragmented placement) may serve as qualitative signals to guide case formulation and monitor change. Finally, because school-based delivery may introduce peer attention or labeling effects, implementation should prioritize privacy protection and minimize unintended social exposure.

The findings should also be interpreted in the Chinese school context, where academic evaluation and high-stakes examinations may intensify stress and shape adolescents’ emotional functioning and help-seeking. In addition, culturally embedded family communication norms and concerns about stigma/privacy may influence both interpersonal trust and participation in school-based counseling, which should be considered in implementation.

From a school-based prevention perspective, brief screening of emotional security, interpersonal trust, and depressive tendency may help identify students who could benefit from early support. Schools may combine universal psychoeducation on emotion regulation and trust-building communication with targeted referral to counseling for at-risk students, while ensuring privacy-protective procedures to reduce labeling effects.

### 4.5 Limitations and future directions

First, the study sample primarily included students from Grades 6 to 8, excluding Grade 9 students who often experience heightened stress due to high school entrance exams, potentially limiting generalizability. Because Grade 9 students face markedly different academic demands and time pressure, the strength and nature of the associations among emotional security, interpersonal trust, and depressive tendencies—as well as responsiveness to school-based interventions—may differ in this group. Future research should explicitly include Grade 9 students and consider stratified sampling or comparing cohorts across key time points (e.g., beginning of term, pre-exam, and post-exam) to examine whether exam-related stress moderates these relationships and intervention effects.

Second, since the sandplay intervention was conducted in a school counseling room, participants’ frequent visits drew attention from peers, which may have influenced their creations. This visibility may have introduced labeling and reactivity effects (e.g., heightened self-consciousness, social desirability, or demand characteristics), which could influence both self-report ratings and sandtray productions. In the present study, sessions were conducted in a private counseling room and confidentiality was emphasized; however, complete concealment of participation in a school setting was not feasible. Future studies should further reduce visibility by scheduling sessions at low-traffic times, using more discrete access procedures, or integrating sandplay activities into universal or classroom-based formats (e.g., whole-class participation) to minimize labeling and peer attention.

Finally, although the study adopted a single-case ABA design (baseline–intervention–withdrawal/follow-up) to strengthen within-participant comparisons, the findings are still limited by the single-participant nature and the constraints inherent to ABA designs. In particular, intervention effects may carry over into the withdrawal/follow-up phase, and observed changes may also be influenced by maturation, history effects, or other concurrent events; therefore, causal conclusions and generalization beyond the case should be drawn cautiously.

Additionally, Study 1 used a cross-sectional design; therefore, the observed associations and the mediation model should be interpreted as correlational/statistical (i.e., an indirect effect) rather than causal, and directionality cannot be established. Longitudinal or experimental studies are needed to test causal pathways among emotional security, interpersonal trust, and depressive tendencies.

Future research should expand the sample to include Grade 9 students and examine the dynamic effects of academic stress on emotional wellbeing across different time points (e.g., beginning of term, pre-exam, post-exam). Intervention settings should also be improved to reduce labeling effects—for example, by integrating sandplay into naturalistic classroom activities or adopting whole-class participation designs.

Methodologically, future studies could combine experimental interventions with cross-lagged models, incorporating family functioning and peer support as ecological variables to build a multilevel causal framework.

## Data Availability

The datasets generated and/or analyzed during the current study are not publicly available due to ethical and privacy restrictions. Further inquiries can be directed to the corresponding author.
