# Supplementary material for: The effect of emotional security on depressive tendencies in junior high school students: mediation and intervention of interpersonal trust
Source: Front Psychol. 2026 Jan 28;16:1736608. doi: 10.3389/fpsyg.2025.1736608 (PMC12891131; doi:10.3389/fpsyg.2025.1736608)
Supplement: Supplementary file 1 [file Table_1.docx]

Appendix S1

### The presentation of the sandbox process

(1) Two individual sandbox sessions during the baseline phase

| **The first sandbox (see Figure 1)** | | |
| --- | --- | --- |
| Date: 2022.5.16 | | **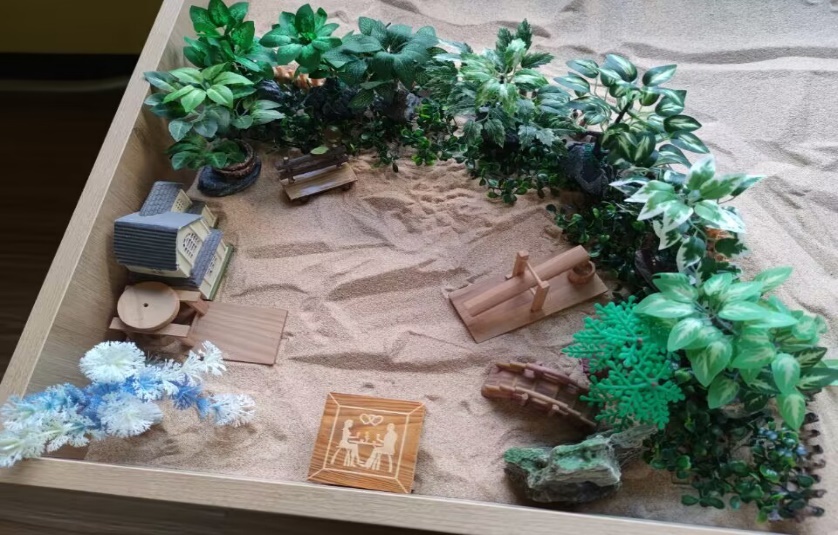**  Figure 1 |
| Subject: None | |  |
| Duration: 11 minutes | |  |
| Self-image: None | |  |
| Use of sand: Undisturbed sand | |  |
| Number of toys: 18 | |  |
| Toy categories: animals, construction | | |
| The creation process and the sandplay scene: When first encountering the sandplay setup, the visitor carefully examined each toy before placing it back, spending considerable time on selection. The overall composition reveals a spacious layout, with the main elements concentrated in the lower left corner of the sandbox. The toys are arranged in a monotonous sequence: surrounding trees, a fence, a rockery, a bridge, a house, blue coral, irrigation tools, a bench, and a mortar. | | |
| The sandbox story: "I wish I could be isolated from the world, surrounded by many trees, yet not sufficiently secluded. It would need to be enclosed by a fence, so even outsiders would know it's a restricted area and not allowed to enter. Then, the other items around the house could allow me to live well on my own, self-sufficiently." | | |
| The counselor concluded: Through the sandplay dialogue, it can be observed that the client yearns for a space of solitude, with interpersonal relationships in crisis, reflecting a closed, negative, and distrustful attitude toward life. The overall composition appears empty, with the artwork occupying one-quarter of the sandplay area, symbolizing the client's inner emptiness and insufficient self-energy. | | |
| **Second sandbox (see Figure 2)** | | |
| Date: 2022.5.23 | 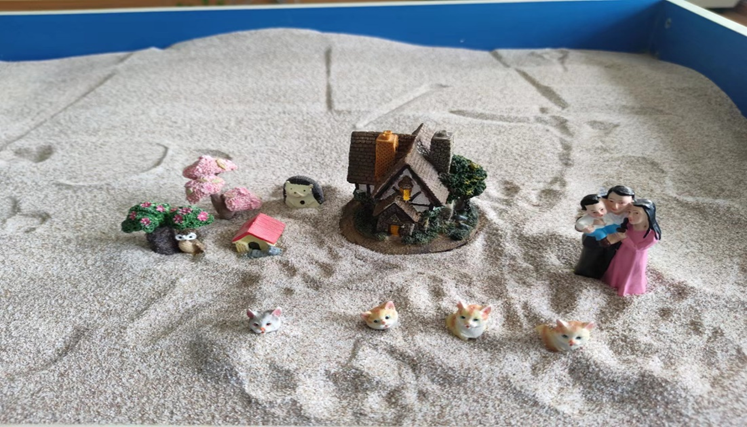  Figure 2 | |
| Subject: None |  |  |
| Duration: 9 minutes |  |  |
| Self-image: little hedgehog |  |  |
| Use of sand: Undisturbed sand |  |  |
| Number of toys: 10 |  |  |
| Toy categories: People, Animals, Plants, Buildings | | |
| The creation process and the sandplay scene: The visitor meticulously selected toys, examining each one before placing them down. Though the composition appears simple, the preparation took considerable time. The sandplay artwork presents an open layout, primarily centered in the lower right corner of the sandbox. The toys are arranged in a monotonous sequence: a large house, a family of three, a little hedgehog, two trees, and a cat's nest, with four kittens buried in the sand at the foreground. | | |
| The sandbox story: "Little hedgehogs love hiding in the sand, avoiding excessive attention and gossip. The kitten's nest in front was where I once kept a cat—it was so adorable, but sadly, I lost it. Those four kittens are now my classmates who keep isolating me. They constantly influence me, surrounding me and making me reluctant to go out." | | |
| The counselor concluded that the sandplay session revealed the client's continued struggle with toxic relationships, marked by distrust and a desire to avoid social interactions. The client sought to minimize attention and commentary. The artwork occupied less than a quarter of the sandbox, featuring small toys and a lack of visual energy. Recent emotional distress manifested as school aversion, with the client preferring home confinement over attending school, perceiving it as a safer environment. This reflects how interpersonal distrust and insecurity can lead to the accumulation of negative emotions. | | |

(2) Eight family sandplay sessions and individual sandplay sessions during the intervention phase

| **First Family Box (see Figure 3** | |
| --- | --- |
| Date: 2022.5.28 | 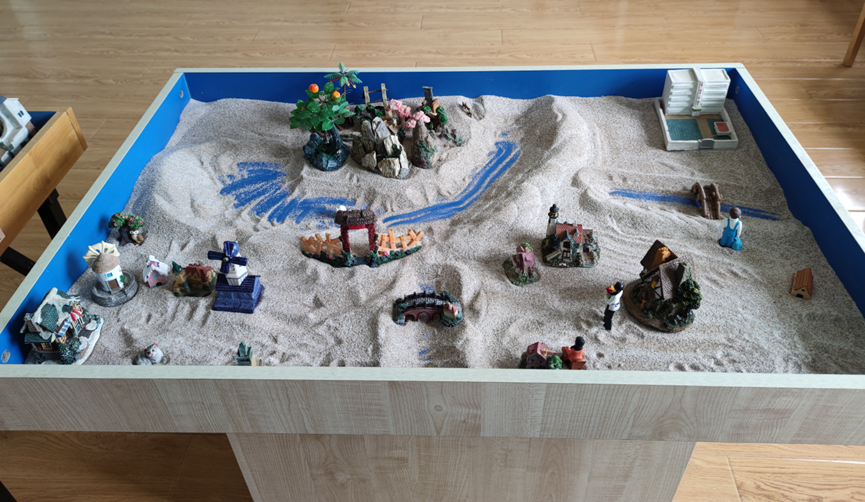  Figure 3 |
| Subject: Land of Hope |  |
| Duration: 33 minutes |  |
| Self-image: A woman sitting on a stool (mother), father-daughter without self-image |  |
| Sand play: sand play between the client and mother |  |
| Number of toys: 26 |  |
| Toy categories: People, Animals, Plants, Buildings | |
| The creation process and the arrangement of the playhouse: Observations revealed that although the rules for creating the family playhouse were explained—requiring each member to take turns after the previous one finished—the visitor's father was always impatient. He would start selecting toys before the visitor did, even placing them down first. During the activity, the three parties occupied separate areas, maintaining a lack of interaction and minimal coordination. The only exception was the mother, who built a bridge in the father's area, and the child, who placed a small gray cat in the father's zone. | |
| Arrangement and layout: Child (upper left), Father (centered in the lower left area), Mother (right side) | |
| The story of the sandbox: Father: 'There's nothing to say. I just want to earn enough money to buy a big house for them to live a good life.'  Mother: "I hope she studies hard and goes to college."  The child said, "That's your own opinion. I'm feeling too tired. I just want my own space."  Consultant: "I noticed an interesting phenomenon—everyone placed toys in their designated areas. The child placed a kitten with the father, while the mother placed a bridge with the father. Could you share your respective feelings about this arrangement?"  The child said, 'His place has no charm at all—just houses. I don't like it, so I just left that cat. We used to have one at home, and he didn't really object.' (The child longs for closeness with her father but feels her parents' strict discipline makes her want to escape.)  Mother: "I built a bridge for you to cross." (Mother plays a role in coordinating communication at home.)  Father: "No particular feelings, just let it go." (Father is a reserved and taciturn figure at home, not particularly good at expressing himself, but he does not oppose family interactions with him.) | |
| The counselor concluded: The initial family sandplay session revealed multiple issues. The artwork clearly demonstrated strained family relationships and structural problems, with toys arranged in a disjointed manner. The analysis identified the mother as anxious and the father as emotionally detached. The child yearned for paternal affection, yet the father showed little responsiveness to her emotional needs. While the mother imposed strict discipline, the client felt stifled and constrained, lacking genuine care. This highlights deficiencies in emotional communication and expression, where members failed to recognize each other's efforts and love. | |
| **Individual box after family box (see Figure 4)** | |
| Date: 2022.5.30 | 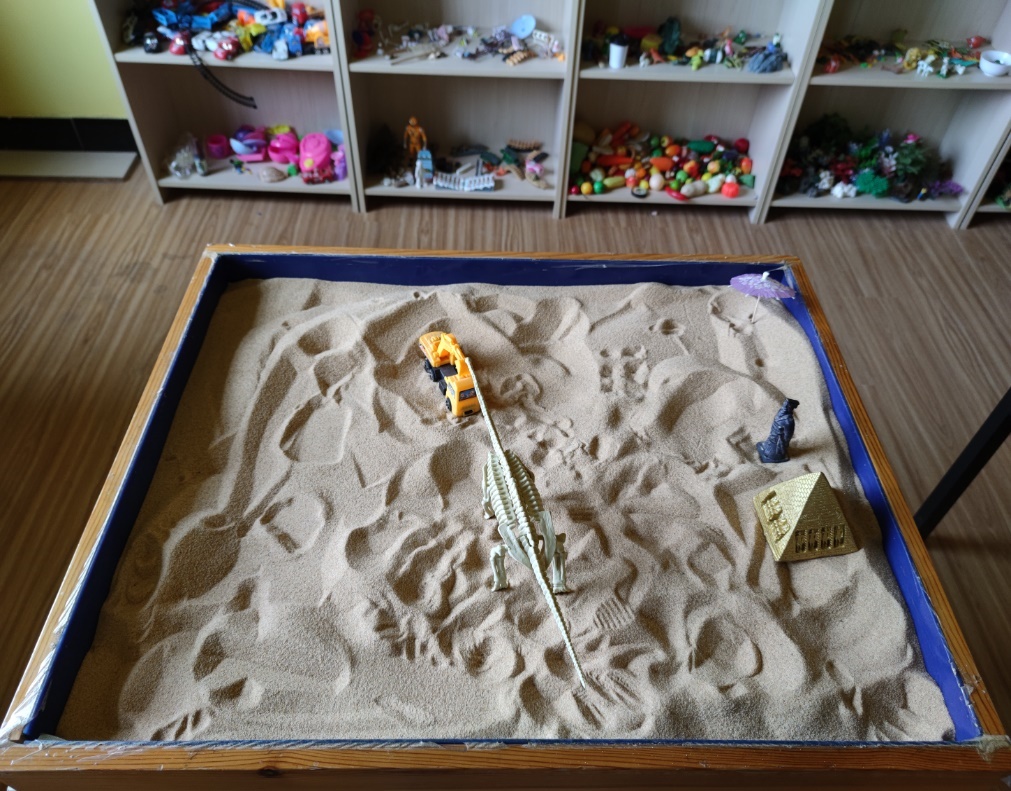  Figure 4 |
| Subject: Childlike Fun |  |
| Duration: 9 minutes |  |
| Self-image: umbrella |  |
| Use of sand: Undisturbed sand |  |
| Number of toys: 5 |  |
| Toy categories: figures, statues, cars, buildings, household items | |
| The production process and the scene of the sandplay: Compared to previous attempts, the creation took significantly less time. An overall analysis of the artwork reveals that the visitor's sandplay composition is notably open, primarily concentrated in the right side of the sandbox. The selection of toys is monotonous, arranged in the following sequence: excavator, dinosaur, pyramid, terracotta warriors, and umbrella. | |
| The sandbox story: "I remember playing sand-digging games alone at my father's shop entrance as a child. This dinosaur skeleton reminds me of how I used to play with two toys colliding—pyramids and terracotta warriors, which I thought represented Eastern and Western cultures. I wanted to see them from a young age. That umbrella, I initially thought, should float in the air, but later placed it in a corner, feeling it was sad (crying)." | |
| The counselor summarized: This time, the client revealed some traumatic events. The toys placed were simple and monotonous, yet conveyed significant information. The toys were concentrated in the center of the sandbox, while the umbrella symbolizing the self remained unplaced, even lying alone in a corner. This reflects the client's childhood, when her parents were often busy with work, leaving her to play alone. She felt deeply lonely and insecure, with almost no friends. This is also a major reason for her lack of interpersonal trust. She perceived her parents and others as indifferent, feeling no affection from them and believing she existed alone. Although she longed to build relationships and seek comfort, she didn't know how to take the first step. | |

| **Second family box (see Figure 5)** | |
| --- | --- |
| Date: 2022.6.4 | 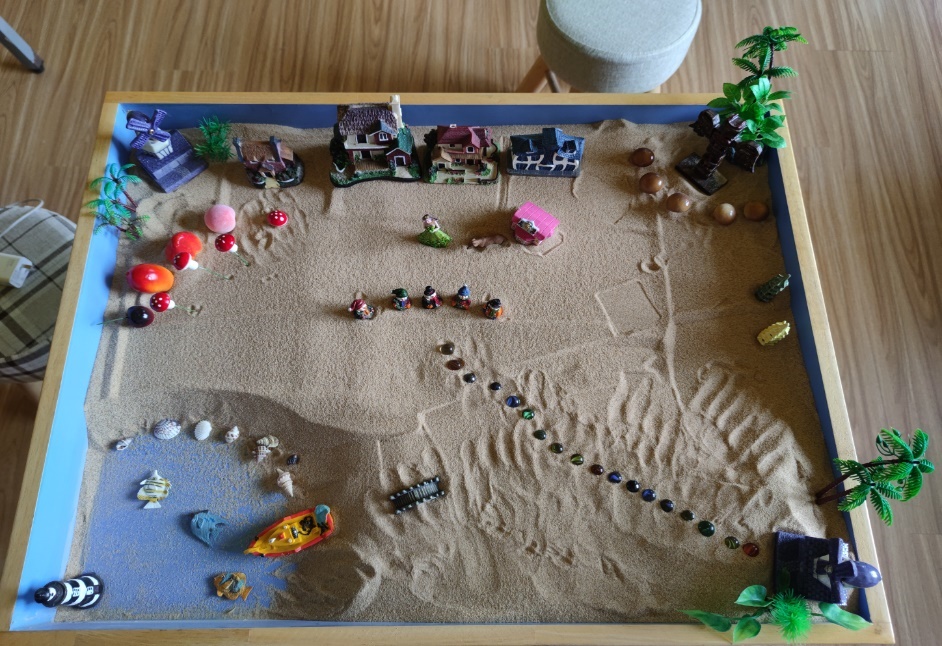  Figure 5 |
| Subject: Home |  |
| Duration: 33 minutes |  |
| Self-image: the woman in the green dress (mother), the lighthouse (female), and the statue in the upper left corner (father) |  |
| Sandplay therapy: the client moves the sand |  |
| Number of toys: 65 |  |
| Toy categories: figures, animals, plants, buildings, vehicles, statues, stones, shells, and other decorations | |
| The creation process and the box garden scene: Observations revealed that the father consistently refined his own works in one designated area, rarely engaging in interactions with others, focusing primarily on the buildings and trees in the upper and lower right corners. The child's interaction with the mother increased compared to previous instances. The overall composition appears more substantial, yet the box garden scene remains fragmented and isolated, lacking coherence and integration. | |
| Placement and layout: Father (focused on the upper area), child (focused on the lower left corner), mother (the central figure and the area that decorates the child and father) | |
| The Story of the Sandplay: Father: "I find the statue of the deity sacred. I am a Buddhist. A house with many rooms feels like home, where a large family can live in harmony."  Mother: "A house needs people too. I've already set up some, plus our stock of fruit."  The child said, "Lighthouses usually light up at night and are seen by people, but they feel lonely. In this sea, only one ship can see it, and they are equally lonely."  Consultant: "I observed the father placing a small bridge on the child's side, while the child placed a kitten and a cat litter box on the mother's side, and two small towers on the father's side. This represents a new addition compared to the previous family sandplay session. Have you noticed this? What are your observations?"  The child said, 'I feel like we're setting up our home. We need a cat, and I love it. '(The child begins to develop a sense of collaborative planning for the home's miniature landscape, gradually stepping out of their own world.)  Mother: "I think it's fine as long as the child likes it." (The mother prioritizes the child's feelings, though she isn't very good at expressing her own emotions.)  Father: "She dug a lake there. I thought a lake should have a bridge. That's all I felt." (The father then decided to communicate with his child and began helping refine his artwork.) | |
| The counselor concluded: The second family sandplay session was still a process of problem exposure. The child and mother interacted more than in the first session, but the mother tended to embellish the child's work, while the father remained in his own area with little involvement from his wife and daughter. This further revealed that in the family, the father was a serious traditional China father, who was not good at smiling, expressing, or communicating. However, during this arrangement, the father and child exhibited positive interpersonal interaction, beginning to unconsciously embellish the child's work, which marked a new breakthrough compared to before. | |

| **Individual box after family box (see Figure 6)** | |
| --- | --- |
| Date: 2022.6.6 | 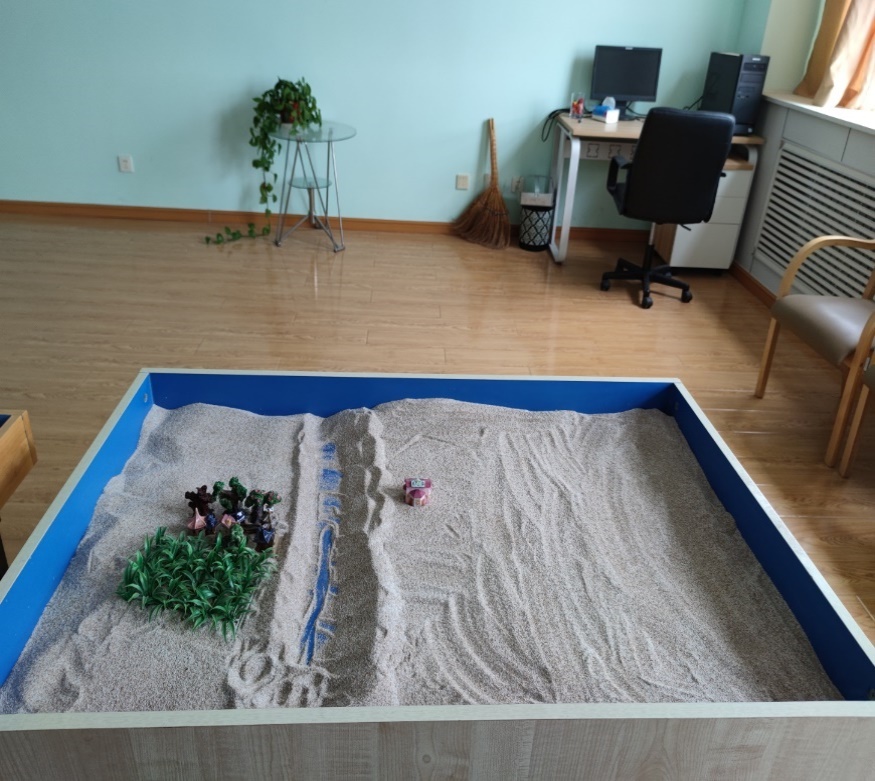  Figure6 |
| Subject: None |  |
| Duration: 9 minutes |  |
| Self-image: None |  |
| Sand usage: dynamic sand |  |
| Number of toys: 10 |  |
| Types of toys: plants, buildings | |
| The creation process and the sandbox scene: The visitor spent less time selecting toys than before, yet hesitated and lingered over placement. While arranging the items, they kept deliberating but ultimately felt unsatisfied with their work, unwilling to spend more time refining it. Analyzing the composition, the overall layout appears spacious, with the main focus on the right side of the sandbox. The toys are selected in a monotonous sequence: a small house, a pavilion, a corridor, trees, grass, a ditch, and a flower shop. | |
| The story of the sandbox: "I want to live in a beautiful place with plenty of greenery, fruit trees, and small animals around. This way, I won't starve, and it won't feel too lonely. There's a flower shop across the street, where I can see fresh flowers displayed in the window every day." | |
| The counselor concluded: The client's toy selection remained monotonous, occupying only a quarter of the sandbox. The enclosed layout, with houses mostly hidden in the woods, reflects the client's withdrawn interpersonal patterns. The flower shop symbolizes a glimmer of warmth and strength in the client's barren inner world. The client hopes for external resources or energy to pull them out of their confined world daily, but currently lacks a sense of safe, trustworthy external support. Thus, they remain distant observers, guarding their hope-filled sanctuary from afar. | |

| **The third family sandplay (see Figure 7)** | |
| --- | --- |
| Date: 2022.6.11 | 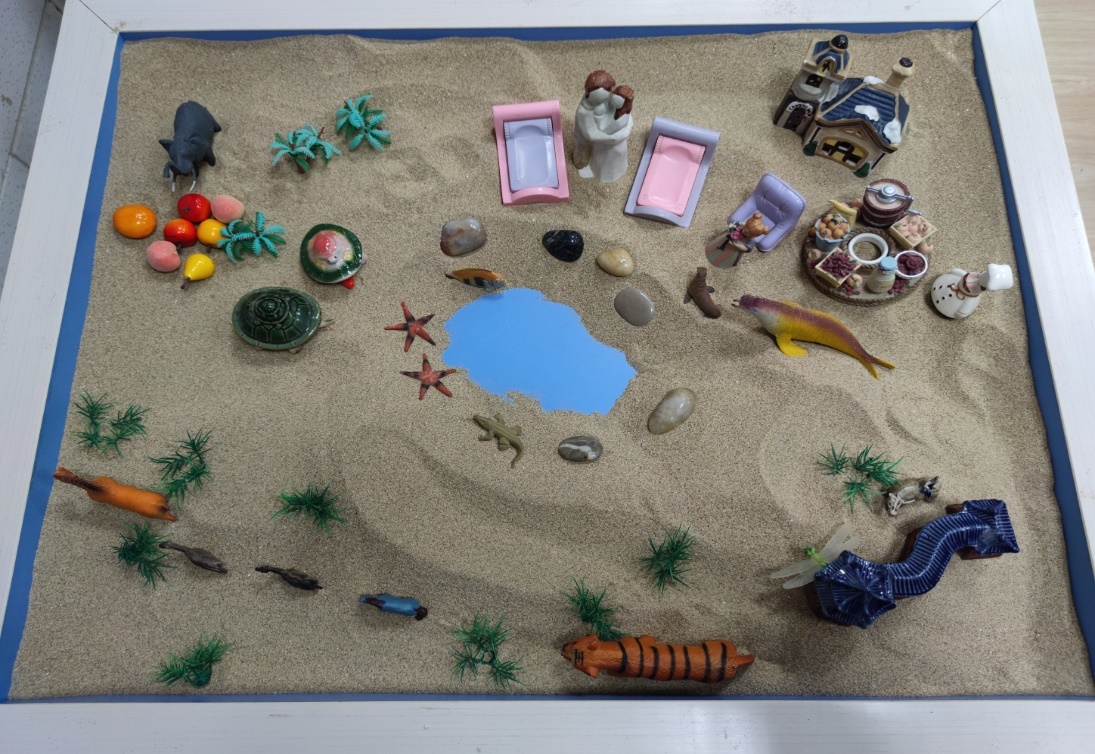  Figure 7 |
| Subject: Life |  |
| Duration: 34 minutes |  |
| Self-image: embracing couple (mother), blue horse (female), tiger (father) |  |
| Sand Usage: Mother's Sand |  |
| Number of toys: 49 |  |
| Toy categories: figures, animals, plants, buildings, marine creatures, etc. | |
| The creation process and the miniature landscape: Several notable conflicts emerged during this project. The child swapped the tiger and blue pony originally placed beside the fruit with the elephant behind them, which the father then returned. The child tried to swap again, but the attempt failed. The child placed a sofa next to the recliner set up by the mother and added a little girl, replacing the mother's stones around the lake with starfish and geckos. While the overall composition was well-rounded, the miniature landscape revealed conflicts and divisions, resulting in a fragmented and disjointed arrangement. | |
| Arrangement and layout: Mother (upper left corner), Father (concentrated in the lower area), Child (more dispersed, initially active in the parents' designated area) | |
| The sandbox story: Father: 'What I'm most pleased with is the lower right corner—it's not just passive but poetic, just like in ancient poems. Other spots are always ruined by this mischievous kid, and my original intention is gone. This child is now so rebellious, always fighting against us (very angry).'  Mother: 'What I love most is how this couple enjoys the lakeside scenery—it's so blissful. Then I noticed the child had added a little girl and a sofa nearby. I thought, "This could be a family picnic!" So I brought tableware, but our cooking turned out terrible. That's why we hired a chef.'  The child said, "They never cared for me since I was little. They only think about themselves. I feel this little girl is actually superfluous. That tiger shouldn't be guarding the food. He should be behind the blue horse, ready to eat the most outcast one."  Consultant: 'During this creative process, I noticed some participants seemed emotionally charged, which led to minor conflicts during the setup. How do you feel about this, or what would you like to convey?'  Child: "I feel redundant. They never cared for me since I was young. When I fell ill, they still thought I was being sentimental. They even beat and scolded me. Whenever they argued, they blamed me. I feel I shouldn't have been born." (The child, deprived of parental affection, experiences an outburst of pent-up emotions.)  Mother: "No, we're too busy with work to play with her every day. When she was younger, she didn't bother us much. But now that she's in junior high and her grades are poor, my husband and I almost accompany her every night to help with her homework. Isn't this what we call caring?" (The mother felt deeply wronged, believing she had sacrificed so much.)  Father: "She used to play with her phone all the time. One night, I saw her still using it and confiscated it. She then cried and screamed, insisting she was suffering from insomnia and depression. What kind of pretense is this?" (The father became emotionally agitated and scolded the child.) | |
| The counselor concluded that the third family sandplay session revealed multiple conflicts and contradictions, particularly in the child's interactions with both parents, where negative interpersonal dynamics were more frequent. The child began to rebel by taking toys placed by the parents, especially in the father's designated area. Through toy arrangement, the child expressed dissatisfaction, perceiving their parents as indifferent and lacking affection. During arguments, the child would mention them, leading to self-blame and the belief that their own actions caused family discord. The child failed to perceive parental love, as the parents' communication style was blame-oriented, resulting in minimal positive parent-child interactions. Once conflicts were fully exposed, the next step involved understanding and empathy to repair the relationship. | |

| **Individual box after family box (see Figure 8)** | |
| --- | --- |
| Date: 2022.6.13 | 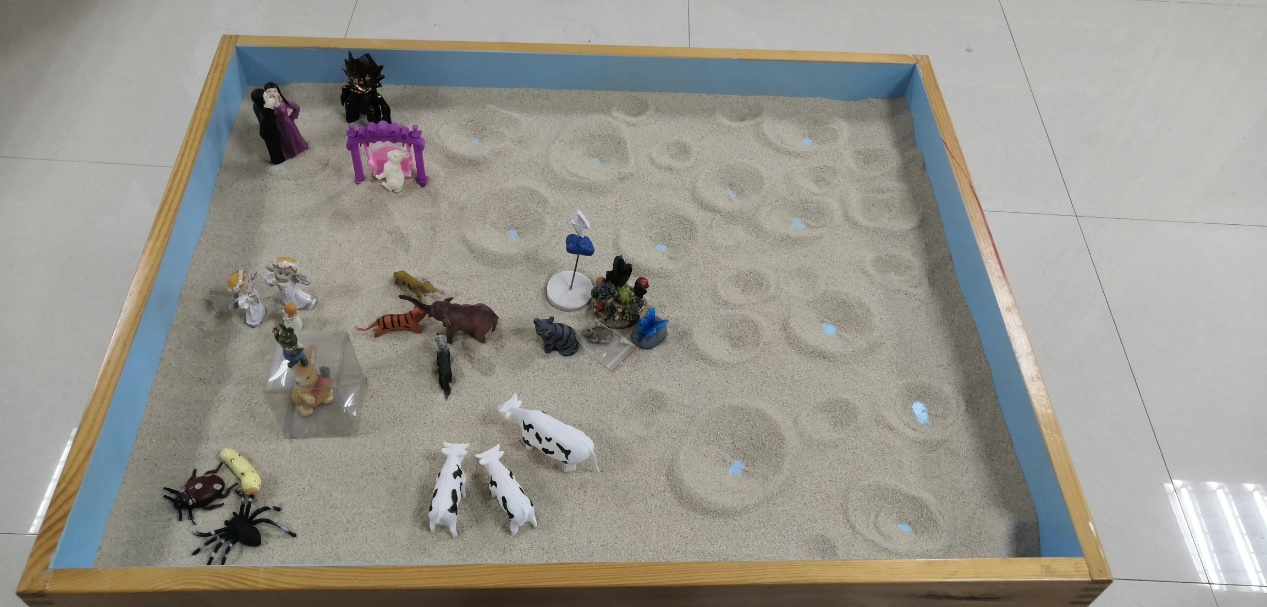  Figure 8 |
| Subject: None |  |
| Duration: 13 minutes |  |
| Self-image: The Rabbit in the Transparent Box |  |
| Sand usage: dynamic sand |  |
| Number of toys: 24 |  |
| Toy categories: animals, figures, insects, street lamps, etc. | |
| The creation process and the miniature landscape scene: During the crafting, the chosen rabbit toys were always encased in transparent plastic, considered a protective measure. While crying, the artist created several puddles, which took considerable time. Analyzing the overall composition, the scene appears desolate and spacious, primarily centered on the left side of the sandbox. The variety of toys expanded compared to earlier works, arranged in the following order: the rabbit in the plastic box, the black old rabbit standing atop it, the gossiping couple, the devil, the swing with the little white rabbit on top, three little angels, two spiders vying for a banana, the elephant surrounded by beasts, the little black cat hiding behind the elephant, the rabbit in the plastic bag, three cows, succulent plants, a blue butterfly, and a streetlight. | |
| The story of the sandbox: "This little rabbit is so pitiful. This place should be its home, and everyone is supposed to protect it. But now, because of some interests, they are fighting. It is now lonely and in danger, and its companions are also threatened. No one can save it, and everyone else just watches its plight with indifference." | |
| The counselor's interpretation: The little rabbit in the transparent box symbolizes the client's inner self-image, reflecting her inner turmoil and the desperate need to break free from constraints for rebirth. In this scenario, the client perceives the plastic box and bag as protective measures for the rabbit, yet simultaneously feels suffocated. While appearing sheltered in a seemingly safe environment, the overall imagery reveals pervasive danger—conflicting interests everywhere. This mirrors her distrust of interpersonal relationships, where she perceives others as self-serving groups with ugly faces, ultimately exposing her sense of powerlessness and emotional suppression toward the current situation. | |

| **The fourth family box (see Figure 9)** | |
| --- | --- |
| Date: 2022.6.18 | 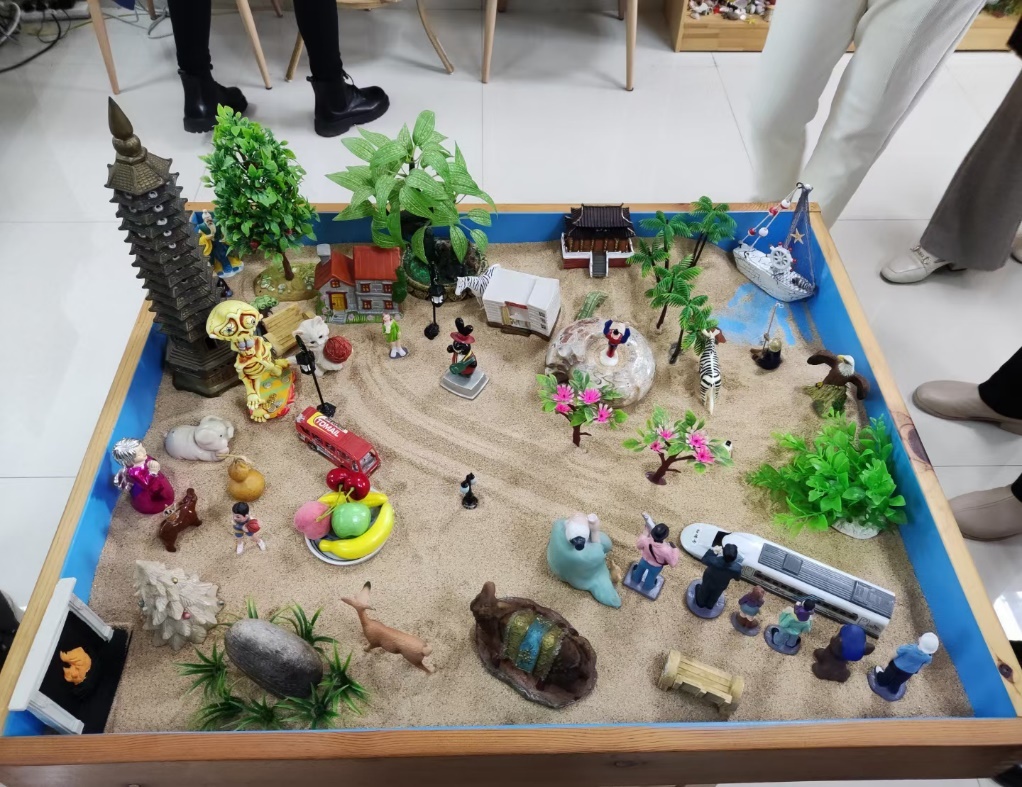  Figure9 |
| Subject: Travel |  |
| Duration: 31 minutes |  |
| Self-image: The little white cat in front of the tower (female), the praying woman (female), the old man fishing (male) |  |
| Sand play: daughter uses fine-grained sand, father uses coarse-grained sand |  |
| Number of toys: 64 |  |
| Toy categories: figures, plants, buildings, skulls, vehicles, stones, seashells, etc. | |
| The creative process and the miniature landscape scene: Conflicts persisted during the creation. The child began placing jarring toys in the artwork, as if silently protesting against the parents 'work. However, the parents stopped removing the toys, even though they were dissatisfied with their placement. Moreover, the clear boundary between the parents' and child's creative zones was blurred. The overall composition no longer appeared fragmented, and the previously disjointed efforts gradually merged. While the artwork showed signs of integration, the presence of contradictions and disharmonious elements remained evident. | |
| Placement order and layout: Child, Father, Mother (each area has its own placement) | |
| The Story of the Sandplay Box: The child said, "I really love this train. I wanted to ride it for a trip, so I drew its path and let it sail into the distance. Then I saw my dad's fishing figure, and I decided to travel to West Lake. There's the Leifeng Pagoda, where a monster's skull sits beneath it. But with him there, the kitten can play freely without anyone daring to bully it. Finally, I added an old man hiding in the woods—he's God watching over everything."  Mother: "I saw the child arrange the train, imagining it packed with passengers ready to depart. Then I envisioned the platform—arranging crowds around it, placing a praying woman near the skull, as if invoking divine protection for their safety. Finally, I realized the train would ultimately reach home, just like how people return for the New Year. At the railway's end, I placed a house, a figure waiting for them to come home."  Father: "When I saw the train, I remembered my past business trips by train. The view outside the window was always mountains, and beyond those peaks lay the sea. I recreated that scene there. The mountain environment was beautiful—there were towering trees and a house where an elderly man lived alone, who loved fishing. I also loved the mountains, water, flowers, and plants, so I placed some of them here and there. But the whole setup felt a bit too sandy and didn't look very appealing."  Consultant: "During this collaborative project, I noticed the team members began working together without prior coordination. However, from your descriptions, it's clear you're making adjustments and completing the work through mutual understanding. Notably, the father gradually joined in on other members' creations, adding floral arrangements and foliage. What are your thoughts on this, or what would you like to share?"  Child: "I feel the big tree, house, and people my parents placed around my tower make it feel awkward—warm yet magical. But when it comes to travel, everyone wants different places, so I can accept it. The most comfortable spot for me is this skull and kitten. Only the kitten knows he's not bad; they're each other's secret friends." (Through his depressive state, the child sends a distress signal to his parents, hoping to gain their love by pretending to be sick.)  Mother: "Everything else is fine, but that skull is just out of place—it's downright eerie. I wanted to get the Buddha statue, but I was afraid my child would object. I believe in Buddhism, but my child strongly dislikes my faith, seeing it as superstitious. The most comforting scenes to me are women praying for everyone's safety, or those waiting anxiously at the doorstep for family to return. It's all so warm and gives me a sense of belonging." (While mindful of her child's feelings, these religious practices provide her with solace during tough times, especially when dealing with marital issues.)  Father: "The most peaceful spot for me is where the angler sits quietly by the lake. I really enjoy this quiet environment, since I'm not much of a social person. This child follows my example too—she often argues with others and ends up fighting when she can't win. I've taught her since childhood that if someone bullies you, you should fight back. Maybe that's why she has so few friends now." (Father began to reflect on himself, sounding somewhat self-critical as he slowly opened up.) | |
| The counselor concluded that while the fourth family sandplay session still contained discordant elements, creating a somewhat contradictory and jarring overall impression, the interpersonal dynamics had shown gradual improvement compared to the third session. Family members began demonstrating cooperative awareness, and the child developed a sense of collaborative creation. The client perceived the skull and kitten as seemingly adversarial yet actually symbiotic guardians. This interpretation revealed the skull symbolizing her depressive state, with the client believing that her perceived 'illness' could serve as an opportunity to receive more parental care and attention. | |

| **Individual box after family box (see Figure 10)** | |
| --- | --- |
| Date: 2022.6.20 | 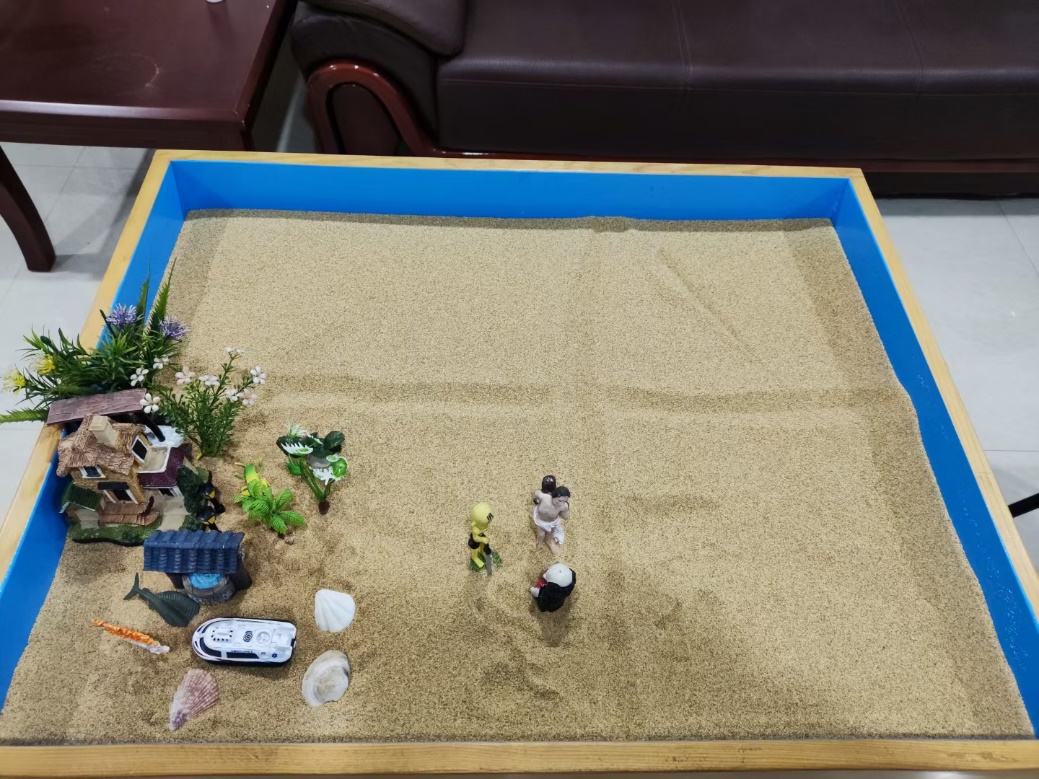  Figure 10 |
| Subject: The Devil Visits |  |
| Duration: 10 minutes |  |
| Self-image: None |  |
| Sand usage: fine sand |  |
| Number of toys: 18 |  |
| Toy categories: figures, devils, plants, insects, vehicles, buildings, shells, etc. | |
| The creation process and sandplay scene: The client first drew an asymmetrical boundary line on the sand. After spending considerable time selecting the well and guardian deity, they remained unsatisfied, believing the well should be dry and the guardian deity should embody both positive and negative elements. Analyzing the overall composition, the scene appears desolate and empty, primarily concentrated in the lower left corner of the sand box. The selection of toys expanded compared to earlier attempts, arranged in the following order: house, flowers and plants, insects behind the plants, well, coral and shells, dead fish, yacht, devil, woman, and guardian deity. | |
| The Sandplay Story: "This land is now parched and water-scarce. The devil invaded the human realm and negotiated with this family, demanding a contract of servitude for rain, forfeiting their freedom. The man wielding a yellow chainsaw is the family's guardian deity, and they refuse to let the devil take over their home." | |
| The counselor concluded: This sandplay artwork reflects the client's inner energy surge, marking her entry into the unconscious realm and exploration of confronting challenges. Though the theme centers on a demon's visit, the guardian deity in the imagery—endowed by the client as a protector of her home—symbolizes safeguarding her inner sanctuary. The absence of water in the well signifies her emotional vulnerability, mirroring her need for a savior to emerge during crises, offering both interpersonal support and strength to help her overcome adversity. | |

| **Fifth Family Box (see Figure 11)** | |
| --- | --- |
| Date: 2022.6.25 | 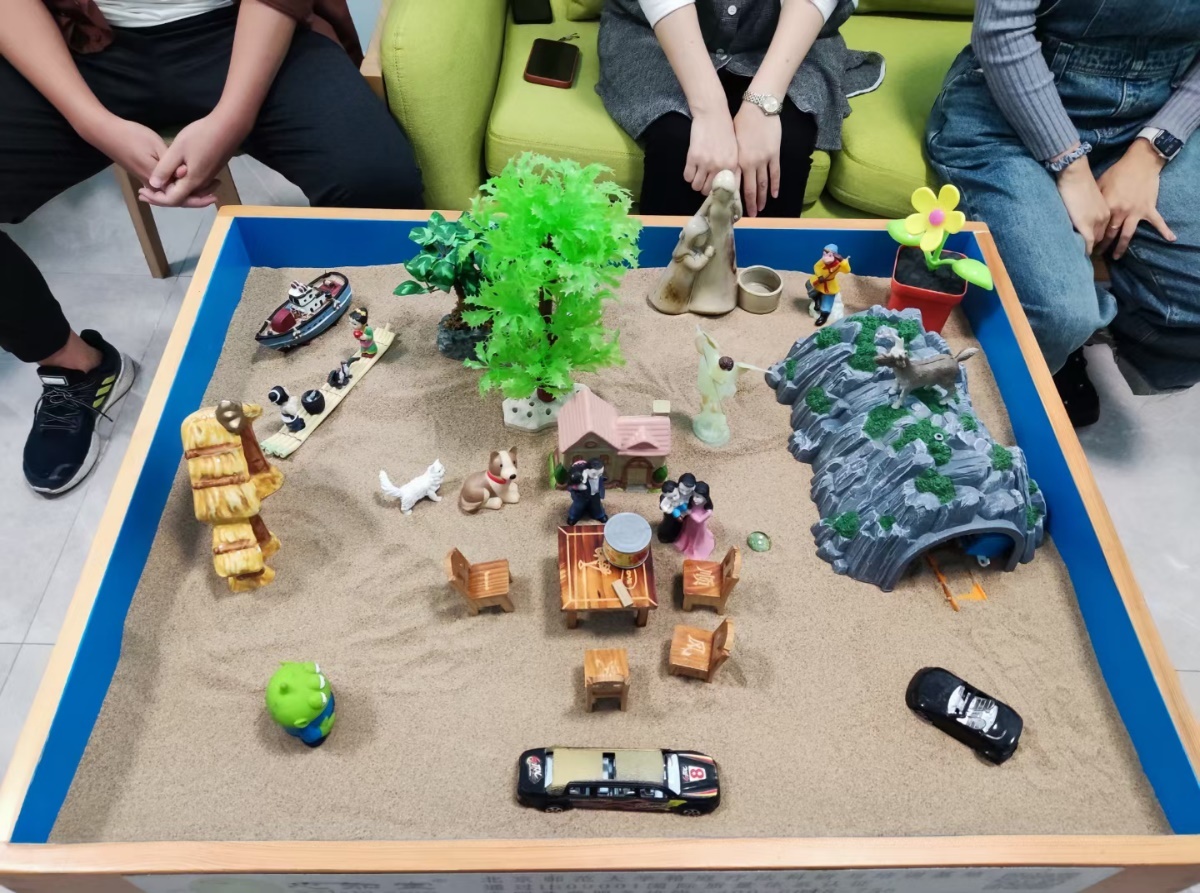  Figure 11 |
| Subject: Vacation |  |
| Duration: 24 minutes |  |
| Self-image: Three-eyed girl (female), a family of three (mother), a man carrying a child (father) |  |
| Use of sand: Undisturbed sand |  |
| Number of toys: 31 |  |
| Toy categories: figures, animals, plants, buildings, vehicles, statues, furniture, etc. | |
| The creative process and the miniature landscape scene: During the creation, conflicts and contradictions ceased to emerge, gradually fostering harmony and stability. The members' mutual understanding improved, and the arrangement of artworks became more relaxed and effortless, free from impatience. The overall composition no longer appeared fragmented, as the previous disjointed approach was replaced by a cohesive trend. | |
| Placement order and layout: Mother, child, father (each area has its own placement) | |
| The Sandbox Story: Child: "When Mom brought this statue upstairs, I knew something was special. I placed a Sun Wukong figurine beside it—after all, he's the ultimate demon-slaying hero with limitless powers. When Dad set up the cave, I added a puppy that barks at passing cars. Then I put a pot of cute flowers and a three-eyed doll. This setup makes the whole space feel so cheerful."  Mother: "I placed a statue of a mother and daughter. It reminded me of how she used to cling to me and play with me as a child—it was so heartwarming. This isn't a deity! The angel was my creation; I find him beautiful and pure. This toy is crystal clear, and I adore it. Later, when I saw the house, I added a family of three in front of it. The two children rowing the boat were also my creations. This kind of life feels so peaceful, like every time I return to my hometown, I don't have to worry about work."  Father: "I originally placed a tree beside the statue, but later realized that where there's a tree, there should be a house. She had a family of three there. Seeing the father carrying his daughter's toy, which looked so heartwarming, I decided to add it too. Then I remembered the train-shaped cave in my hometown with similar features, so I recreated it. In the end, I added a car to let the family travel around."  Consultant: 'During this creative project, I noticed how you all worked together—when someone put down a toy, others would either adjust it or add another toy of the same type. What did you feel about this, or what would you like to share?'  Child: (Silence, no response)  Mother: "I think this scene is so bright and warm, with a strong sense of home. It feels like celebrating a festival back home—everyone rarely argues or has disagreements, and it's really heartwarming." (The mother noticed her child's genuine cooperation and positive transformation)  Father: "I also think this time is pretty good. It's the lifestyle I really enjoy." | |
| The counselor concluded: The fifth family sandplay session demonstrated a clear trend toward integration, with members engaging in more positive interactions while negative ones nearly disappeared. The increased interaction between the child and father reflected the father's positive transformation—showing greater attention to the child's emotions and behaviors. The child, feeling the father's love, became more proactive and cooperative. The family's rapport improved significantly, marking a qualitative breakthrough in the parent-child relationship during this session. | |

| **Individual box after family box (see Figure 12)** | |
| --- | --- |
| Date: 2022.6.27 | 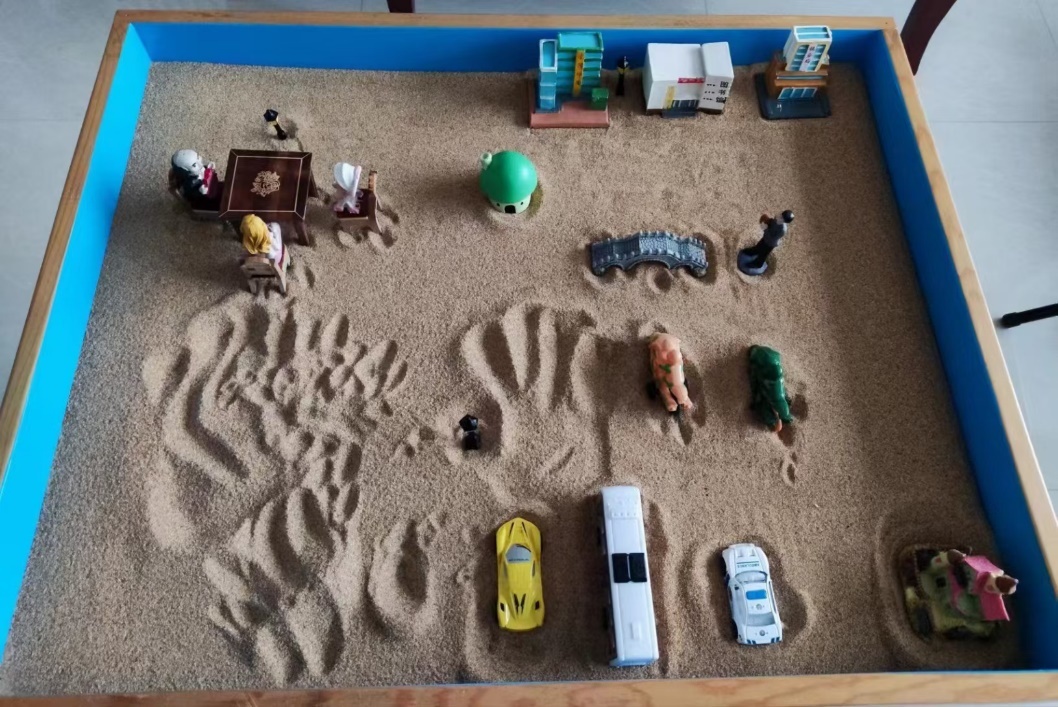  Figure 12 |
| Topic: Negotiation with the Devil |  |
| Duration: 13 minutes |  |
| Self-image: The little girl on the stool |  |
| Sand usage: fine sand |  |
| Number of toys: 21 |  |
| Toy categories: figures, devils, vehicles, buildings, street lamps, furniture, sculptures, etc. | |
| The creation process and the miniature landscape scene: During the creation, the artist first imprints intricate patterns on the palm, then arranges the sand toys. The initial expression of furrowed brows gradually relaxes, and the toys are placed with deliberate pressure, seemingly imbued with emotional intent. Analyzing the composition, the overall scene appears desolate yet expansive, featuring a diverse array of toys. These toys are progressively endowed with symbolic meanings of interpersonal relationships, trust, and communication. The arrangement follows this sequence: table and chair, demon, woman and child, street lamp, small green house, small bridge, father, two fierce beasts, car, and the house in the lower right corner. | |
| The Story of the Sandplay: "It was a deep night when the devil lured the mother and daughter into his house for a negotiation. The father was about to rescue them, needing to cross a bridge with an abyss and wild beasts below. I couldn't tell if he would succeed. The ground bore many dragging marks, yet their conversation didn't feel tense—it was more like a casual chat in the courtyard." | |
| The counselor summarized: This theme resonates with the previous individual sandplay session, reflecting the visitor's growing inner energy, primarily manifested in the following aspects: First, regarding self-representation, figures representing the visitor begin to emerge. Though still in their childhood with underdeveloped wings, these figures become more concrete, supported by protective elements. Second, the father's image plays a pivotal role in this artwork. For the first time, the father participates in the rescue efforts. Despite facing challenges, he begins to show concern for the visitor, actively helping them through their difficulties. This reflects increased real-life interactions between the visitor and father, as they work together to overcome obstacles, elevating the father's status in the visitor's heart. Finally, the visitor describes the negotiation scene with the devil as peaceful rather than tense, indirectly indicating that family members and the visitor are now willing to communicate and solve problems together, rather than arguing or blaming each other. | |

| **The sixth family box (see Figure 13)** | |
| --- | --- |
| Date: 2022.7.2 | 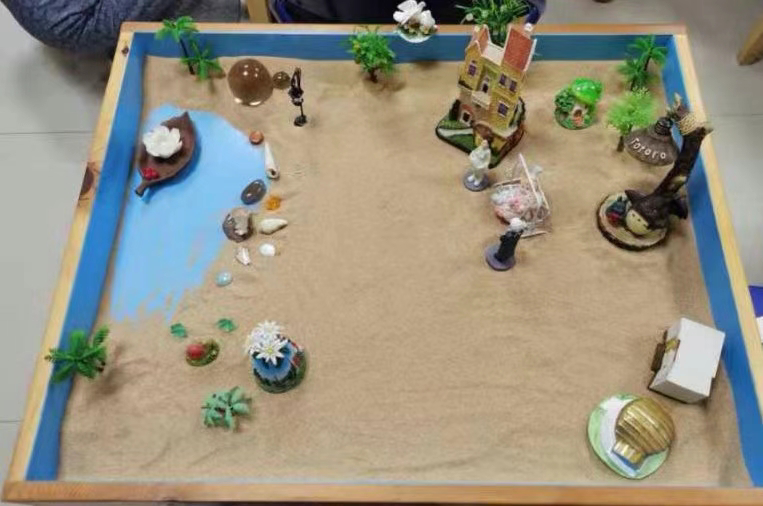  Figure 13 |
| Subject: A Quiet Harbor |  |
| Duration: 23 minutes |  |
| Self-image: The woman on the swing (mother), the house of green mushrooms (female), the white lotus on the boat (father) |  |
| Sand usage: Father moves the sand. |  |
| Number of toys: 36 |  |
| Toy categories: figures, plants, buildings, seashells, etc. | |
| The production process and the miniature landscape scene: Compared to previous attempts, the child actively participated in and refined the parents' artwork, with family members making subtle adjustments to enhance the overall coherence and harmony of the composition, gradually developing a sense of the bigger picture. The overall composition is more substantial, with the miniature landscape scene demonstrating a trend toward coordination and integration, resulting in a harmonious and serene visual effect. | |
| Placement order and layout: Father, Mother, Child (each area has its own placement) | |
| The Story of the Sandpit: Father: "I fell in love with this little boat adorned with white lotus flowers at first sight, but I wanted to place it in the lake. So I began by digging a lake, then planted some greenery that matched the setting. When I saw the house and library built by her mother and daughter, I added some trees and decorative elements around them."  Mother: "I want to place some small pebbles by the lake, like the cobblestone paths in park lakeside areas. Then I can see a small boat. I'll set up a rattan chair there for a view of the lakeside, and install streetlights for illumination. The library needs to be somewhere, since there's no space on the right."  Child: "I placed the crystal ball by the lake. Though lakes usually hold treasures, this one's too shallow, so I had to leave it on the shore. Then I lit the Totoro lamp for nighttime illumination. Around the rattan chair, I gathered my family. That green mushroom house is truly magical—it holds a spell that helps me forget my troubles. But it blends in with the trees, so it's hard to spot."  Consultant: "I would like to ask, would you allow your parents or friends to discover this green mushroom house?"  Child: "I don't think my parents are a problem, but I'm a bit uneasy about my friends. I don't have any real friends—they always laugh at me. I don't want to face them at school, and I don't want them to enter my secret hideout." (The child displays positive energy, with self-repair abilities emerging and trust in parents growing. However, trust in friends still requires further openness.) | |
| The counselor concluded: The sixth family sandplay session demonstrated growing integration in the overall composition, with enhanced coordination among family members. The client actively participated in refining their parents' creations. Their self-image was visualized as a green mushroom house, which they considered a secret sanctuary to escape worries. They expressed willingness to share this space with their parents, reflecting the basic restoration of a harmonious parent-child relationship characterized by mutual trust, sharing, and communication. However, the client remained hesitant to open up to others, fearing potential harm or school-based interactions. Further intervention is still required to establish trust in interpersonal relationships. | |

| **Individual box after family box (see Figure 14)** | |
| --- | --- |
| Date: 2022.7.4 | 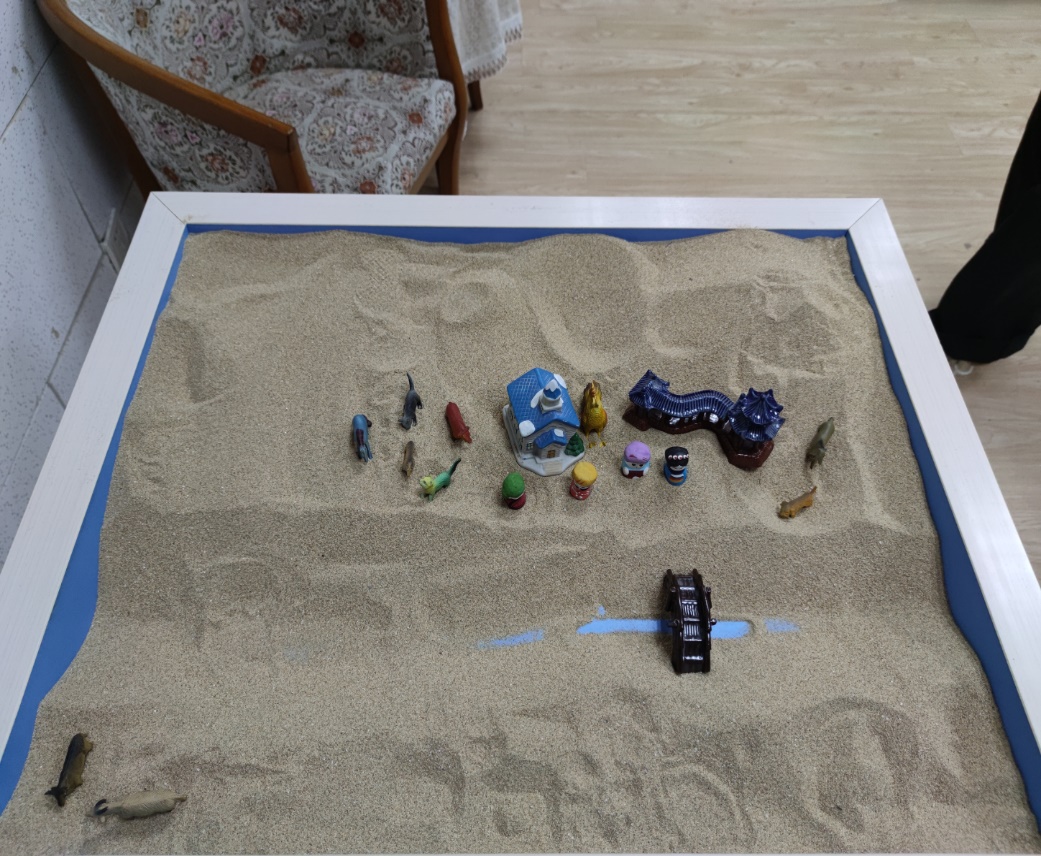  Figure 14 |
| Subject: None |  |
| Duration: 12 minutes |  |
| Self-image: Four little people in front of the house |  |
| Sand usage: dynamic sand |  |
| Number of toys: 18 |  |
| Toy categories: People, Animals, Buildings | |
| The creation process and sandplay scene: The visitor first drew a small stream on the sand and placed toys in the center, talking to themselves. The overall composition appears desolate and empty, with the main focus on the central area of the sand box. The selection of toys is monotonous, arranged in the following order: a house, a rooster, four little figures, a small pavilion, two animals nearby, two puppies, a bridge, and an animal in the lower left corner. | |
| The story of the sandbox: "I envisioned a harmonious coexistence between humans and animals. In front of the house, a river flows with a small bridge, allowing animals to cross to the opposite bank and humans to reach the other side. Everyone lives in harmony, with no one infringing upon another." | |
| The counselor summarized: In this individual sandplay session, we observed the client's self evolving from a singular figure to multiple representations, akin to the four figures in front of a house. These figures can be interpreted as the client, her family, and her closest friends. The sandplay imagery reflects her desire and bold attempts to build harmonious interpersonal relationships. Although the scene lacks fullness and energy, the client imbued the toys with meanings related to interpersonal trust. For instance, the small bridge spanning the two sides connects different areas, facilitating communication and mutual assistance between people and animals. The presence of animal-shaped toys in groups also demonstrates the client's transition from a closed, independent space to an open, symbiotic environment. | |

| **The seventh family box (see Figure 15)** | |
| --- | --- |
| Date: 2022.7.16 | 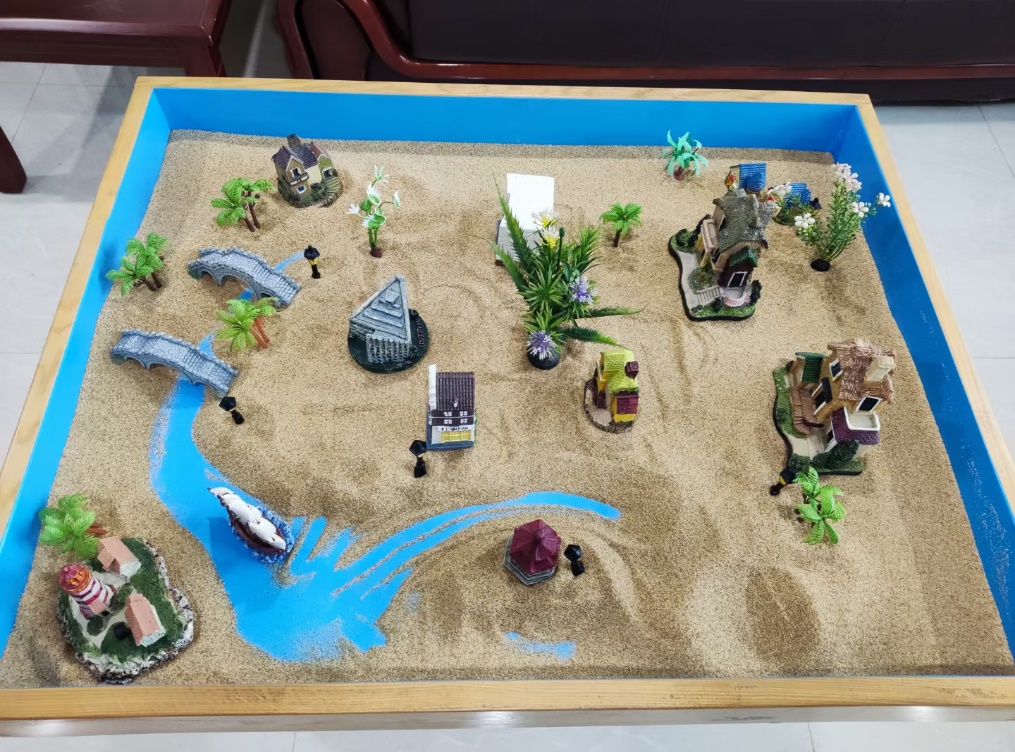  Figure 15 |
| Subject: Park |  |
| Duration: 38 minutes |  |
| Self-image: none |  |
| Sand play: The client and father play with sand, while the mother gently manipulates the sand. |  |
| Number of toys: 29 |  |
| Toy categories: Plants, Buildings, Vehicles, Streetlights, and other decorations | |
| The production process and the miniature landscape scene: During the production, the members demonstrated higher levels of tacit understanding and coordination, with family members working together to ensure the overall coherence and harmony of the composition. The final image appears substantial, with the miniature landscape scene exhibiting a sense of coordination and integration, resulting in a harmonious and serene overall visual effect. | |
| Placement order and layout: Father, child, mother (each area has its own placement) | |
| The Story of the Sandplay Box: Father: 'This time, I wanted to create a scene of a small bridge over flowing water and a house. I started by placing a house with green plants, then designed the river's flow, and finally arranged several large houses to define each location. I made sure every house had windows that opened to reveal the beautiful scenery outside.'  The child said, 'When I saw my dad digging the river, I knew he was trying to create a certain scene. With a river, I thought a small boat, a bridge, and a pavilion would be perfect for it. It instantly evoked the charm of a Jiangnan water town.'  Mother: 'I noticed the house they had built, so I added streetlights, flowers, and trees to the small bridge. I thought this would make the environment more beautiful and pleasant.'  Consultant: 'From your description, it seems you were mentally hypothesizing the other party's ideas during this project, collaborating based on their concepts rather than working independently. Is that correct? Do you have any specific messages you'd like to convey about this?'  My child said, 'I don't think I like to go against them anymore. I used to hate my dad's design ideas—they felt so lifeless. Now I think it's great when we all work together to make things happen.'  Mother: 'We can clearly see how our child has changed, and we've gradually become more attuned to each other's thoughts. This arrangement feels a bit more natural now.'  Father: "I've also noticed significant changes in my child, who now communicates willingly with me. Although we didn't discuss this beforehand, the arrangements we made were all satisfactory, and I find it quite positive." | |
| The counselor concluded: The seventh family sandplay session presented a more harmonious, complete, and substantial overall composition, demonstrating a strong trend toward visual integration. Positive interpersonal interactions among family members were both frequent and well-coordinated. The child and father engaged in frequent positive interactions, revealing that when a father takes the initiative to change, it triggers significant transformations in the family structure and relationships. This highlights the father's crucial role in children's education and interpersonal development. However, most traditional fathers are stereotyped as stern, unemotional, and often neglectful. Yet during a child's growth, a father's companionship, encouragement, and support are vital for building self-confidence, self-esteem, and psychological resilience. | |

| **Individual box after family box (see Figure 16)** | |
| --- | --- |
| Date: 2022.7.11 | 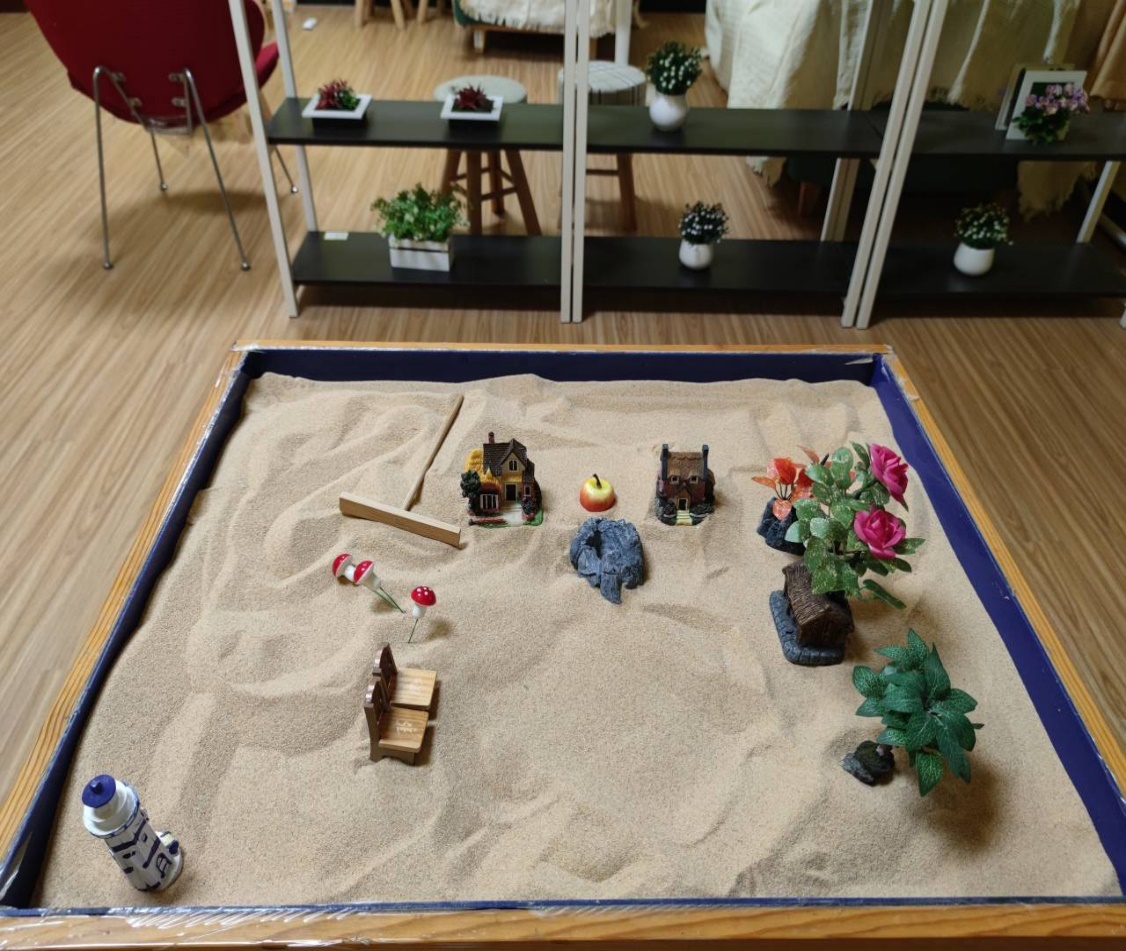  Figure 16 |
| Subject: Rescue |  |
| Duration: 10 minutes |  |
| Self-image: The Man in the Rockery |  |
| Use of sand: Undisturbed sand |  |
| Number of toys: 13 |  |
| Toy categories: Figures, Plants, Buildings | |
| The creation process and the miniature landscape scene: The visitor first spent considerable time leveling the sand with sand tools, but quickly selected toys. Analyzing the overall composition, the scene appears spacious. Although the variety of toys is limited, some symbolic items representing self-recovery and interpersonal relationships are present. The arrangement follows this sequence: house, apple, figurine, rockery, tree, followed by another house, chair, mushroom, and lighthouse on the right. | |
| The story of the sandbox: "A person was trapped beneath the mountain. I thought of Sun Wukong, who had endured years of hardship under the sun and wind. At last, he met someone who could rescue him and set him free. The world beyond is truly wonderful. He would wait for that person to appear, then watch the sunrise and sunset together in the courtyard. From that moment on, his world would be filled with colors." | |
| The counselor concluded: While the seventh individual sandplay session featured fewer toys and a minimalist, empty composition, it revealed the client's pent-up energy. The client chose a figure in the artificial hill as their self-image, likening it to Sun Wukong (the Monkey King) trapped beneath the mountain, awaiting Tang Monk's rescue to brave hardships and explore the beautiful world beyond. This reflects the client's transition from isolation to seeking a companion through adversity, embracing the vision of a shared journey to discover the world's wonders. Symbols like the lighthouse, trees, and fruits in the composition—representing hope and vitality—highlight the client's inner richness and the theme of rebirth. At this stage, the client demonstrates a qualitative leap compared to earlier phases. | |

| **The eighth family box (see Figure 17)** | |
| --- | --- |
| Date: 2022.7.16 | 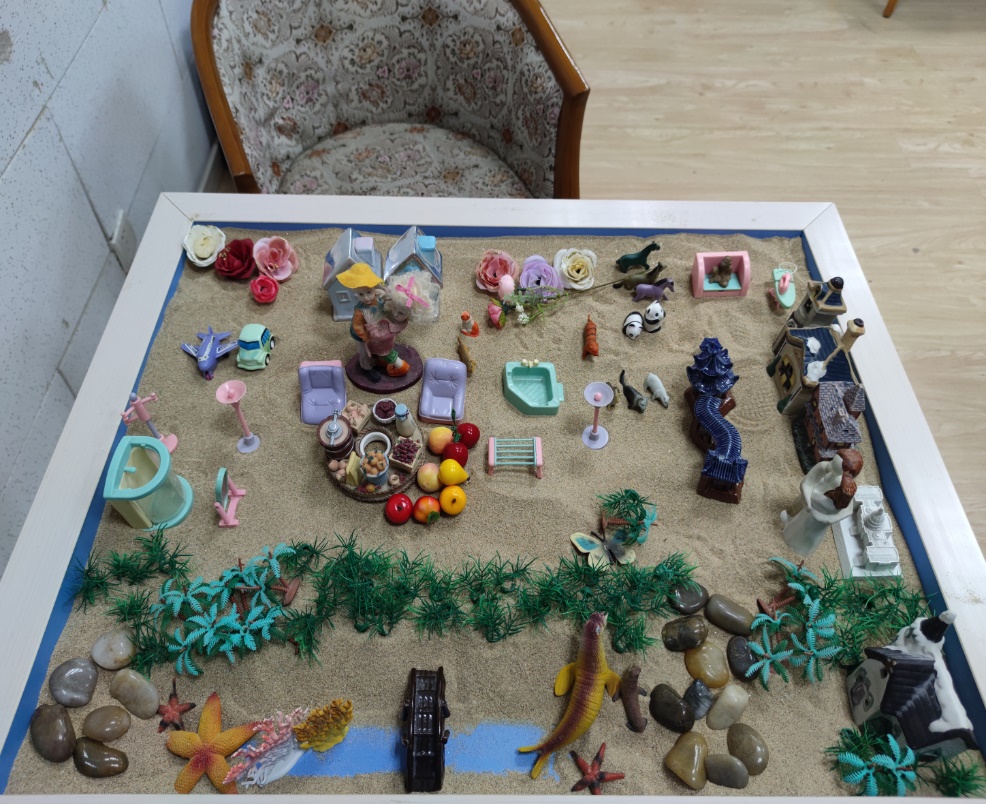  Figure 17 |
| Subject: Sweet Home |  |
| Duration: 38 minutes |  |
| Self-image: a sculpture of embracing (mother), a friend embracing (female), and a house in the upper right corner (father) |  |
| Sandplay therapy: the client moves the sand |  |
| Number of toys: 66 |  |
| Types of toys: animals, plants, buildings, vehicles, statues, stones, seashells, and other decorations | |
| The production process and the miniature landscape scene: During the production, the members demonstrated higher levels of tacit understanding and coordination, with family members working together to ensure the overall coherence and harmony of the composition. The overall image is substantial, with the miniature landscape scene exhibiting strong coordination and integration, featuring abundant energy flow, resulting in a harmonious and serene visual effect. | |
| Placement order and layout: Child, Father, Mother (each area has its own placement) | |
| The Sandbox Story: Child: "I picked up those two sparkling little houses. They looked so beautiful, and I imagined them as my family's home. The two cuddly kids seemed as close and friendly as the Haier brothers, sharing a deep bond. Then I saw Mom setting up a recliner for them, so I dug a little river in front of it, creating a scene of a small bridge over flowing water and a cozy home. After that, I placed a fruit tray, adding a touch of vacation vibes. Finally, I added some adorable little animals—small and harmless, perfect companions for humans."  Father: "I noticed the child had placed a house in the center, so I thought I'd put mine beside it. After adding several houses, I saw my daughter's artificial lake and planted some greenery. In the end, I thought this little pavilion was lovely—so poetic, symbolizing that life isn't just about the mundane but also about poetry and distant horizons, haha."  Mother: "I placed this embracing statue in front of my son's father's house. It makes me feel that no matter how far away, parents will always look toward their children and care for them. Then I arranged some stones and marine creatures as decorations by the lake, which reminds me of a summer beach. I also placed some fruits beside my daughter's dining table so she won't be picky about food."  Consultant: "I noticed the mother has placed numerous daily necessities and food items in the child's designated area, with the figures positioned there consistently gazing toward the child. The father is also engaged in decorating the child's space. Moreover, your house is oriented toward the child, which clearly demonstrates your attention and care for them. Do you have any thoughts or messages regarding this?"  The child said, "Now I understand a bit more and can feel their care for me. Even though they sometimes yell at me, at least I know they love me and want me to be well."  Mother: "There are no parents who do not love their own children. It's just that we don't know what you need and only want to provide you with the same material foundation as other children. Being busy earning money might lead to a lack of psychological attention for you."  Father: "I've been restraining my temper lately and trying not to hit or scold her. The child has grown up and is different from when she was little. It's hard to discipline her now, sigh." | |
| The counselor summarized: The final family sandplay session presented a richly layered and harmonious composition, radiating energy. Each member's thoughtful arrangement demonstrated exceptional teamwork and coordination, resulting in a cohesive visual presentation. As the intervention nears its conclusion, the post-session discussion and reflection revealed that family members actively shared their thoughts and feelings in a harmonious and joyful manner, requiring minimal guidance from the counselor. Throughout this process, significant progress was observed in communication, mutual support, sharing, and understanding. Clients began to heed their parents' advice and comprehend their decisions, while parents themselves showed marked changes—starting to prioritize their children's emotional needs. This led to improved parent-child relationships, strengthened basic trust, and the gradual establishment of secure and reliable trust with others. | |

| **Individual box after family box (see Figure 18)** | |
| --- | --- |
| Date: 2022.7.18 | 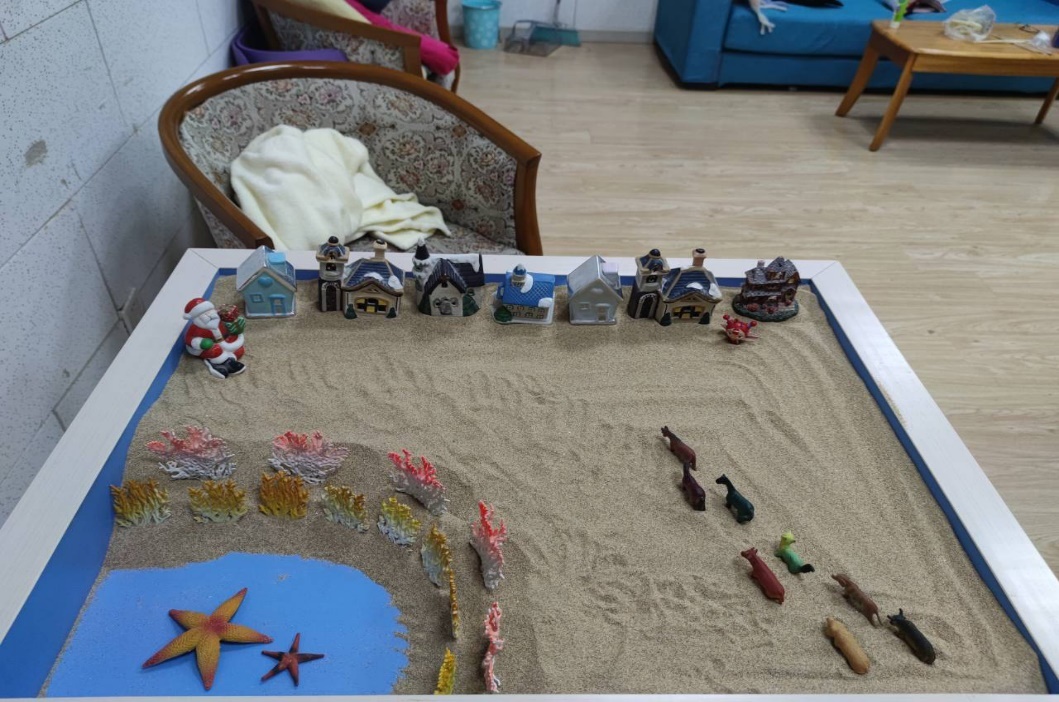  Figure 18 |
| Subject: Christmas |  |
| Duration: 15 minutes |  |
| Self-image: Elf |  |
| Sand usage: dynamic sand |  |
| Number of toys: 33 |  |
| Toy categories: animals, plants, figures, buildings, starfish, corals, and other decorations | |
| The creation process and the miniature landscape scene: The visitor first dug a deep pit, meticulously collecting excess sand and carefully selecting colorful small animals and houses. This painstaking process took considerable time. Analyzing the composition, the scene appears richly detailed, featuring toys symbolizing self-recovery, self-discovery, and interpersonal relationships. The arrangement follows this sequence: coral, starfish, small animals, houses, Santa Claus, and elves. | |
| The Sandplay Story: "This is a Christmas-themed story. During the holiday season, the little elf invites his friends to visit his village. Flocks of animals gather in the town, bringing festive cheer and lively energy. Nearby, a lake holds a mysterious treasure hidden among the coral reefs. This area remains shrouded in mystery, known only to me and my teacher." | |
| The counselor summarized that the final individual sandplay session showed several notable changes compared to previous sessions: Firstly, the selection of toys was more diverse and abundant. Secondly, the scenes were more vivid and positive, with toys symbolizing interpersonal communication and interaction appearing more frequently. Thirdly, the client began actively exploring their subconscious, gaining a clearer understanding of themselves, and gradually establishing a source of inner energy. In the final summary and reflection, the client mentioned their recent social interactions, noting that with the support of family and friends, their depressive symptoms had significantly improved. This demonstrates the crucial role of interpersonal trust and social support systems in alleviating depression. | |

(3) Six Individual Sandbox Tracing Sessions

| **The first individual sandbox (see Figure 19)** | |
| --- | --- |
| Date: 2022.8.29 | 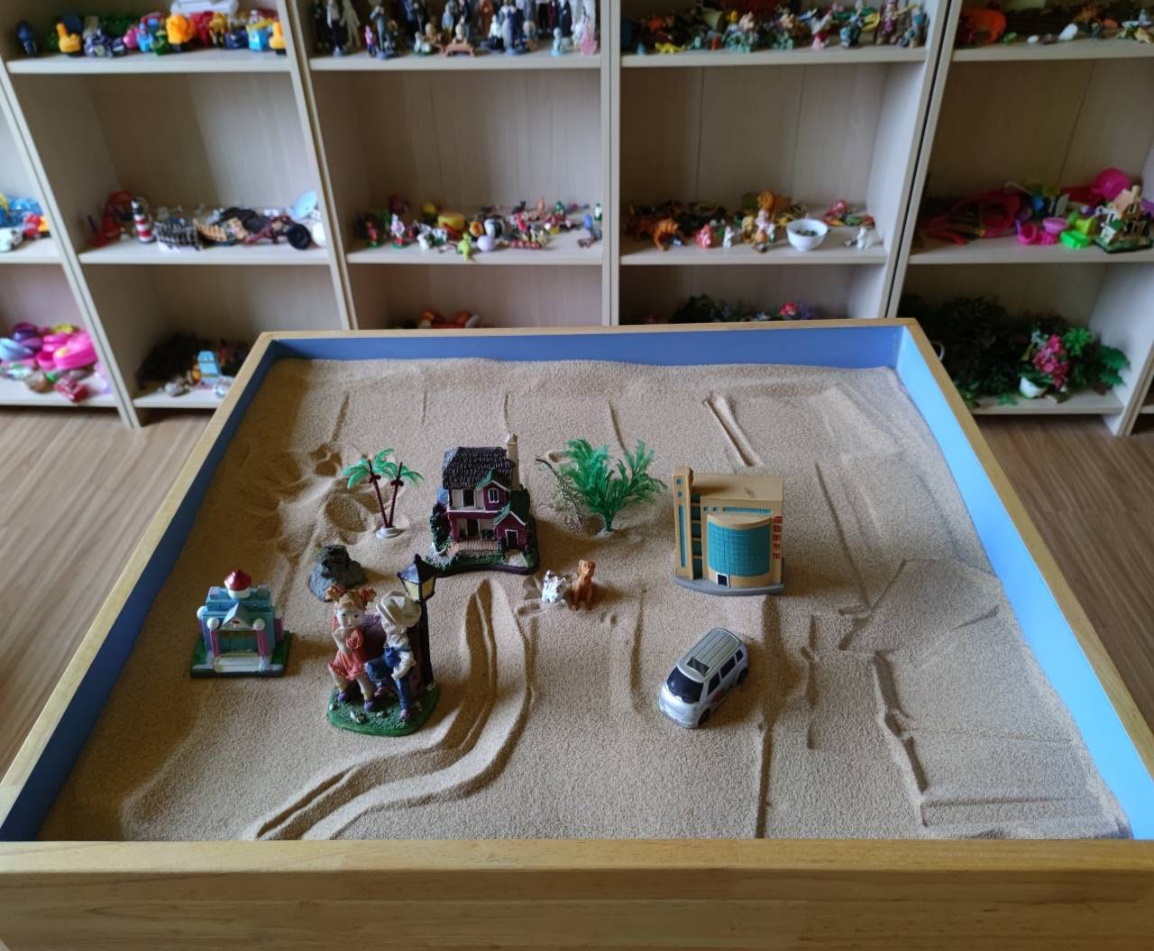  Figure 19 |
| Subject: My Future |  |
| Duration: 14 minutes |  |
| Self-image: Two little people |  |
| Sand usage: fine sand |  |
| Number of toys: 10 |  |
| Toy categories: People, Animals, Plants, Buildings, Vehicles | |
| The sandplay process and scene: The client first placed a house at the center of the sandbox, then drew a winding path across the sand. When selecting toys, they hesitated about the library option, repeatedly picking it up and putting it down. The overall composition appears well-balanced. Though the limited selection of toys reflects future planning, it reveals the client's aspirations and hopes for the future, moving beyond confusion or avoidance. The toys are arranged in the following order: the central house, a small tree, the library, a figure, a cat and dog, a rockery, a blue house, and a car. | |
| The sandbox story: "When the school year began and I entered the graduating class, my teacher asked us to reflect on our future aspirations. She said the high school entrance exam would be a turning point. I decided to create a theme about my future—this is what my life might look like in ten years: following my parents 'wishes, I'll get into college, find a partner, own a car, and have a house with a cat and a dog. During holidays, we can take road trips." | |
| The counselor summarized: During a one-on-one sandplay session in the follow-up phase, the client, who had just started the new semester and entered the graduating class, was experiencing significant academic pressure. The theme was chosen based on recent events. The client discussed their future plans, selecting toys and themes that were positive and uplifting. They depicted two interdependent figures, reflecting their hopeful aspirations and future plans, marking a shift from the previous avoidance of such topics. | |

| **The second individual sandbox (see Figure 20)** | |
| --- | --- |
| Date: 2022.9.26 | 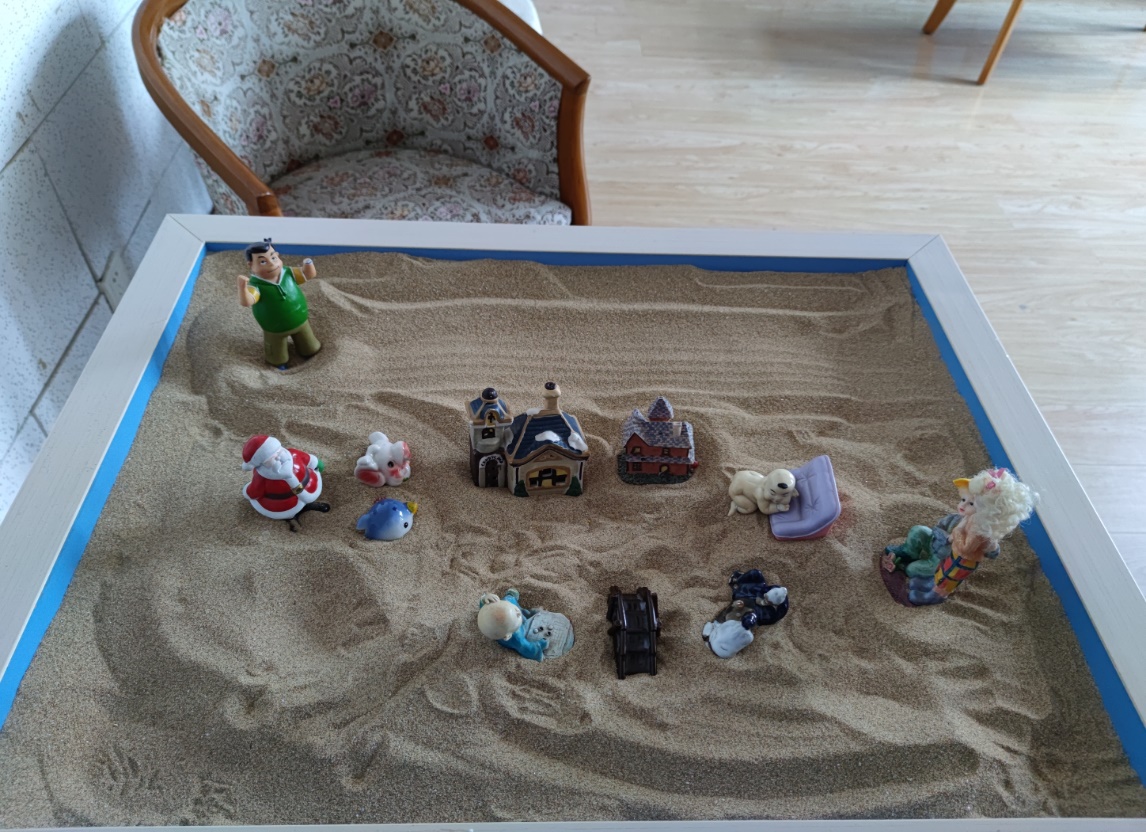  Figure 20 |
| Subject: Simple |  |
| Duration: 16 minutes |  |
| Self-image: The child on the sofa |  |
| Sand usage: fine sand |  |
| Number of toys: 12 |  |
| Types of toys: sculptures, figures, buildings, small bridges, etc. | |
| The creation process and miniature landscape scene: The most time-consuming elements were selecting a child representing the creator and the guardian deity, the green-clad boy, while other toys required less time for selection and production. Analyzing the composition, the overall arrangement appears well-balanced and dynamic, featuring a diverse array of toys. The sequence of placement follows this order: a small house, a sofa, a child, a ceramic doll, Santa Claus, parents, a small bridge, an elderly chess player, a young monk, and the green-clad boy. | |
| The sandbox story: "This was my childhood home scene before the Lunar New Year. My parents and grandparents lived close by. Grandpa would play chess with my uncle, while I played on the sofa. My parents were busy cooking, and those two crystal-clear toys were my piggy banks. During the New Year, I could fill them up. The Santa Claus and piggy banks placed side by side looked especially adorable. I once had an identical ceramic version at home. The child resembling Monk Isshu was my younger brother, who was very smart and loved by everyone. The little boy in green was the family guardian, a strongman who watched over us in the corner, giving us a sense of security." | |
| The counselor concluded: During the second follow-up session in the individual sandplay therapy, the client arranged their favorite aspects of life. The self was depicted as a child on a sofa, reminiscing about the warm and joyful atmosphere of the New Year celebrations at their grandparents 'home. The client placed a Hercules figurine as a guardian deity, noting that guardian figures frequently appeared in their creations. This reflects the client's longing for a strong and reliable father figure to protect them and their mother, seeking security from the father. In this harmonious setting, the client experienced the warmth of home and felt inner peace and contentment. | |

| **The third individual sandbox (see Figure 21)** | |
| --- | --- |
| Date: 2022.10.31 | 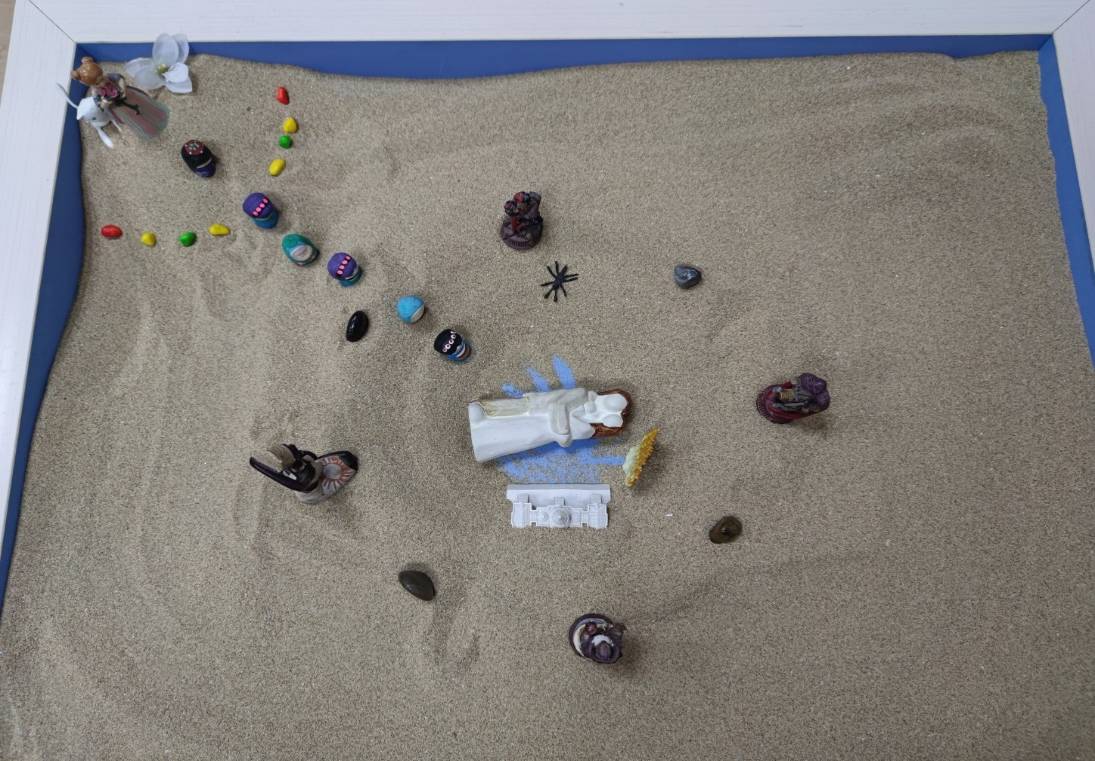  Figure 21 |
| Subject: Redemption |  |
| Duration: 22 minutes |  |
| Self-image: two people embracing each other |  |
| Sand usage: dynamic sand |  |
| Number of toys: 29 |  |
| Toy categories: figures, plants, insects, buildings, statues, stones, and decorations such as corals | |
| The creation process and the sandbox scene: Given the abundance of sand in this sandbox, the visitor spent considerable time digging the base layer, yet still felt it lacked depth. They then added decorative elements to create an impression of a deep-sea abyss, dedicating significant effort to selecting the divine statues. Analyzing the composition as a whole, the scene appears rich and dynamic, with a vibrant energy flow. The selection of toys is diverse, arranged in the following sequence: two embracing figures, a palace, coral, black stones, spiders, divine statues, angels, a super dream, flowers, colorful pebbles, and figurines. | |
| The story of the sandbox: "When I saw these two people embracing, I was suddenly reminded of a scene from the depths of the ocean—where they were hugging in a palace, facing the gods' judgment. In the heavens, an angel carrying a dream sent other little angels to the abyss to rescue them." | |
| The counselor concluded that during the third follow-up session in the individual sandplay therapy, the client's inner world had undergone significant changes. The client revealed minor conflicts with classmates and teachers at school, yet found companionship during their period of emotional distress. This sense of support and encouragement manifested in the artwork as two figures embracing each other under divine judgment. However, they received interpersonal support and assistance, with the little angels dispatching companions to rescue them. Thus, although the characters appear to be in difficulty, the external energy and support are abundant, breaking through the predicament and allowing energy to flow freely. This reflects the client's positive transformation in both emotional state and interpersonal support compared to previous periods. | |

| **The fourth individual sandbox (see Figure 22)** | |
| --- | --- |
| Date: 2022.12.5 | 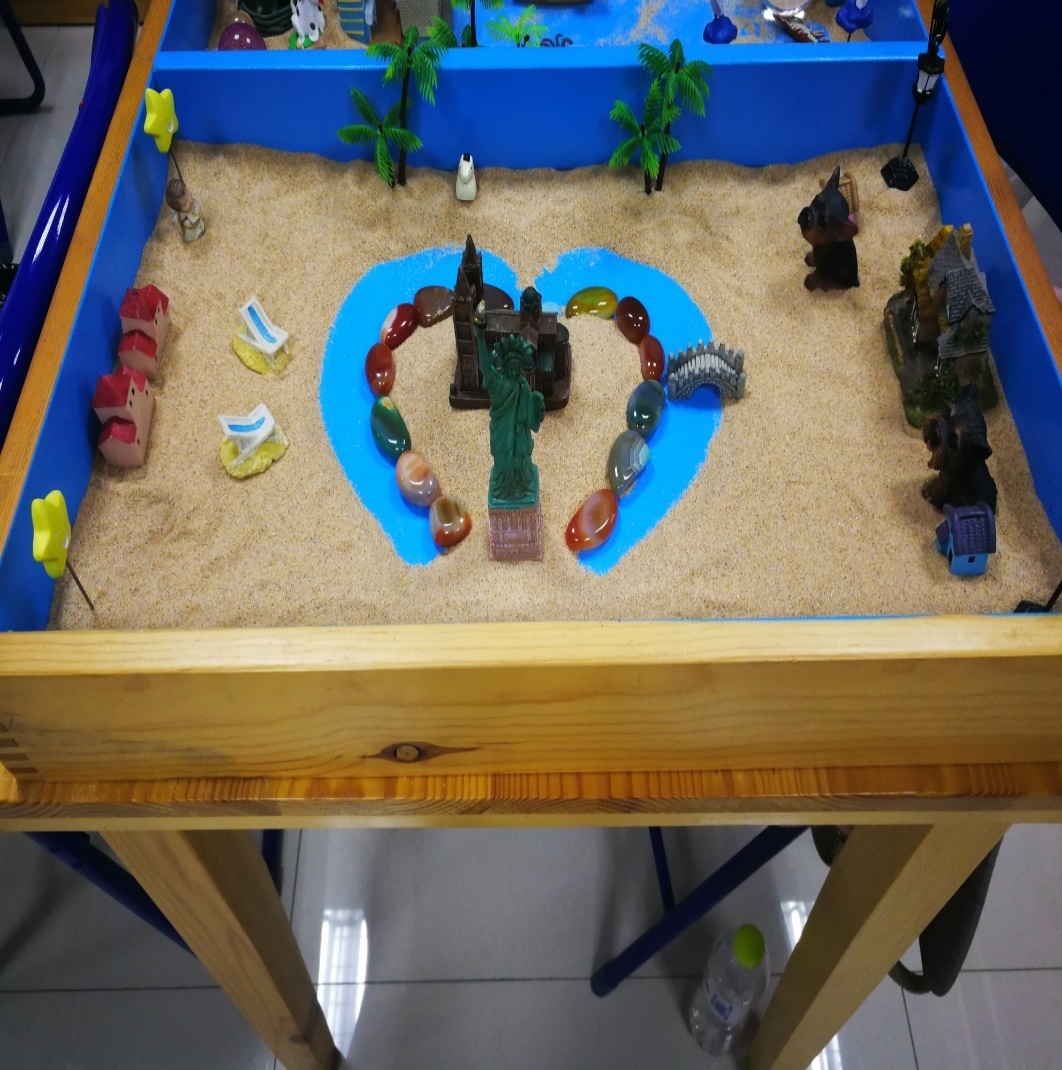  Figure 22 |
| Subject: Long live freedom! |  |
| Duration: 21 minutes |  |
| Self-image: Statue of Liberty |  |
| Sand usage: dynamic sand |  |
| Number of toys: 32 |  |
| Types of toys: animals, plants, buildings, vehicles, statues, stones, street lamps, and other decorations | |
| The creation process and the miniature landscape scene: The visitor first built a small island in the center using sand, then dug a small river around it to form a heart-shaped island. During the toy selection phase, they chose brightly colored and warm-toned toys, spending considerable time on this step. Analyzing the overall composition, the scene appears rich and full, with a sense of energy flowing through it. The selection of toys is diverse, arranged in the following order: stones on the island, a house, the Statue of Liberty, a recliner, a small house, a streetlight, a small tree, a pony, a puppy and its kennel, and a small bridge. | |
| The sandbox story: "I yearn for freedom and cherish its value. This week, my mindset has transformed profoundly—I've discovered my life's purpose. In this world I've crafted, everyone thrives with joy and freedom. Here, you can vacation, explore, and even amass your own fortune." | |
| The counselor's summary: During the fourth follow-up session in the individual sandplay therapy, the client reported having emerged from a period of confusion and low spirits, gaining clarity about life and embracing a self-directed existence. Her chosen artwork—a towering and dynamic Statue of Liberty—reflects her inner energy and emotional fulfillment. Through this session, combined with the client's account, it was evident that she had established a strong interpersonal trust, exhibited minimal depressive symptoms, and developed a rational and stable self-concept along with psychological resilience. | |
| **The fifth individual sandbox (see Figure 23)** | |
| Date: 2023.1.9 | 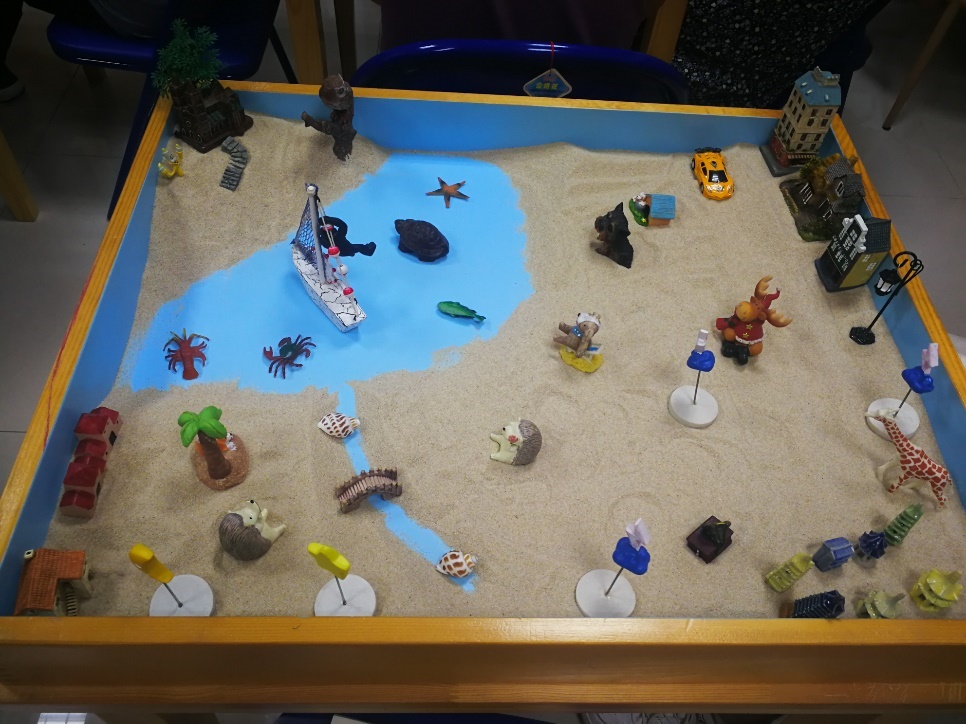  Figure 23 |
| Topic: The Town of Fairy Tales |  |
| Duration: 22 minutes |  |
| Self-image: little hedgehog |  |
| Sand usage: dynamic sand |  |
| Number of toys: 48 |  |
| Types of toys: animals, plants, buildings, vehicles, statues, stones, seashells, and other decorations | |
| The sandplay process and the miniature landscape: The child first piled sand to form a small hill, with the sea below. Throughout the activity, the child remained cheerful, humming a tune. The overall composition is rich and dynamic, with a flow of energy. The toys are carefully arranged in sequence: a house on the hill, a candle, a path, a small house in the upper right corner, a tower and streetlight in the lower right corner, a hedgehog, a bridge, a giraffe, a deer, a puppy, a recliner, a sailboat, a chimpanzee, and seafood in the lower left corner, along with a house and a tree in the lower left corner. | |
| The Story of the Sandpit: "This is a fairy-tale world where an ancient castle perches atop a towering mountain, housing a treasure trove. Adventurous gorillas often sail to explore, returning with all sorts of peculiar finds. The village's two little hedgehogs meet daily on the bridge to chat and share gossip. The young deer serves as the village chief, guarding the settlement. The giraffe, standing tall and mighty, offers a telescope-like vantage point to survey the castle." | |
| The counselor noted that during the fifth follow-up session, the client's thematic imagery in the individual sandplay became significantly more intricate and fantastical. The client's choice of two hedgehogs gazing across a small bridge as their self-portrait reflected their positive interpersonal dynamics, particularly in sharing and communication. The castle on the mountain symbolized the client's aspirations for the future, while the sailboat and gorilla representing wealth acquisition highlighted their well-planned life goals and abundant self-energization. The recurring guardian deity motif also revealed the client's reverence for and longing for their father, expressing a desire for paternal protection to provide security and fulfillment. | |

| **The sixth individual sandbox (see Figure 24)** | |
| --- | --- |
| Date: 2023.2.13 | 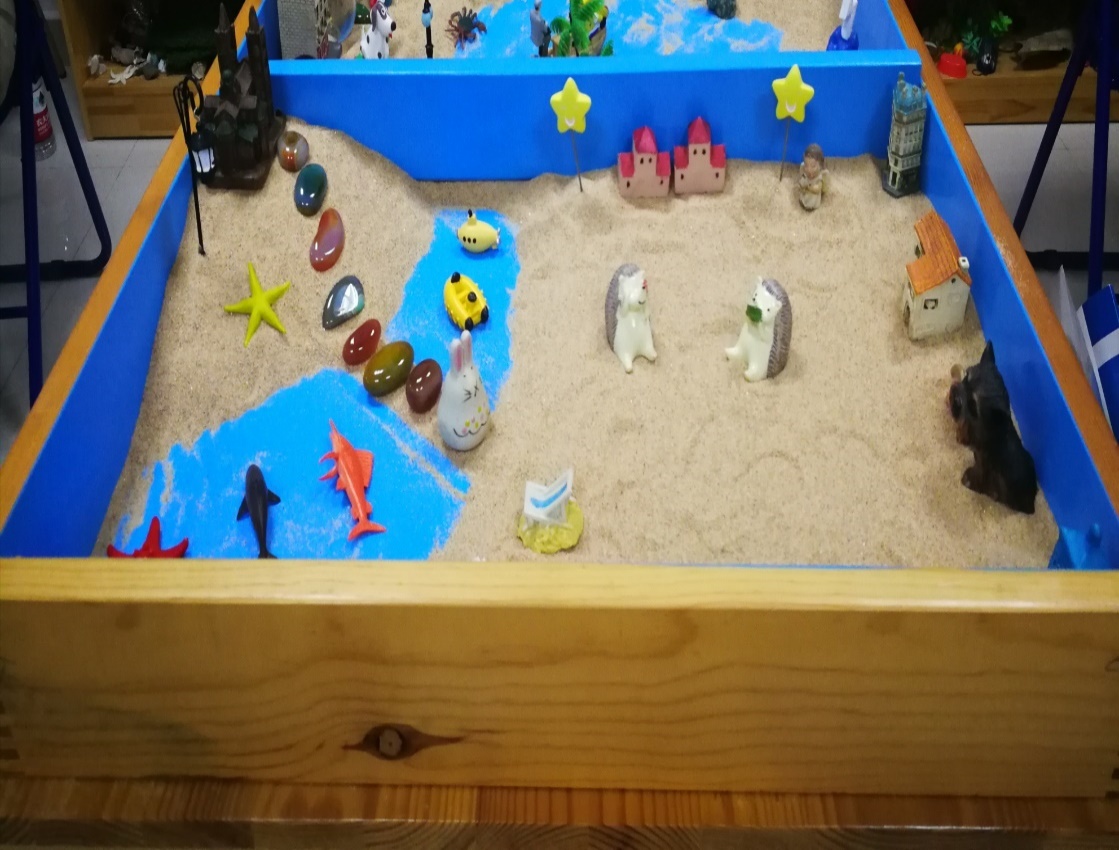  Figure 24 |
| Subject: Park |  |
| Duration: 18 minutes |  |
| Self-image: little rabbit |  |
| Sand usage: dynamic sand |  |
| Number of toys: 28 |  |
| Toy categories: animals, streetlights, buildings, stones, starfish, etc. | |
| The creation process and the miniature landscape scene: The visitor first builds a sand hill, with a lake at its base separated by a descending path. Their creativity and imagination flourish as they reinterpret toys with new meanings and uses. The composition is rich and dynamic, featuring a diverse array of toys arranged in order: a house on the hill, streetlights, stones, a little rabbit, a hedgehog, a recliner, a small house, stars, an angel, a black dog, fish, a rubber boat, and a starfish. | |
| The Story of the Sandplay Box: "This time, I want to create a charming and cozy theme. This is their happy planet. Every day, the little rabbit comes down from the mountain castle to play with the little hedgehogs. This area is their paradise, free from any disturbance or worries." | |
| The counselor concluded: In the sixth follow-up session's individual sandplay therapy, the client's self-image transformed into a rabbit descending a mountain, now resembling the enigmatic castle atop the peak. This signifies the client's clearer future planning and methodical implementation, with more extensive pathways and vessels leading to the other shore. The streetlight in the imagery symbolizes energy, illumination, and directional guidance, reflecting the client's inner attainment of spiritual integration and healing, aligning with the future while refining and developing their selfhood and personality. | |
